# Comparison of outcomes after living and deceased donor kidney transplantation: UK national cohort study

**DOI:** 10.1093/bjs/znaf162

**Published:** 2025-08-19

**Authors:** James Murray, Annabel Luke, David Wallace, Chris Callaghan, Linda D Sharples

**Affiliations:** Department of Medical Statistics, London School of Hygiene and Tropical Medicine, London, UK; Department of Medical Statistics, London School of Hygiene and Tropical Medicine, London, UK; Department of Medical Statistics, London School of Hygiene and Tropical Medicine, London, UK; Department of Hepatology and Liver Transplant, The Royal Free Hospital, NHS Foundation Trust, London, UK; Department of Nephrology and Transplantation, Guy’s Hospital, Guy's and St Thomas’ NHS Foundation Trust, London, UK; Department of Medical Statistics, London School of Hygiene and Tropical Medicine, London, UK

## Abstract

**Background:**

Most kidneys for transplantation come from deceased donors, though healthy live individuals may also donate. Living donor transplants generally show better outcomes than deceased donor transplants, but it is unclear whether this reflects inherent benefits of having a living donor kidney or differences in donor and recipient characteristics. Using data from 10 915 UK kidney-only transplants, the aim of this study was to determine the causal effect of living donors on graft survival, considering all-cause death without graft failure as a competing risk.

**Methods:**

This study used inverse probability of treatment weighting based on propensity scores to adjust for imbalances in baseline variables between recipients of living and deceased donor kidneys implanted between 2010 and 2021. The mean treatment effect, had all patients received kidneys from living donors, was estimated from differences in survival probabilities and restricted mean survival time using weighted competing risks models.

**Results:**

After adjustment for key confounders, living donor kidney transplantation (LDKT) was associated with a 6.03% (95% c.i. 4.71% to 7.35%) lower 5-year risk of graft failure compared with deceased donor kidney transplantation (DDKT). Over 7 years, living donor recipients experienced an additional 0.36 (95% c.i. 0.29 to 0.43) years of graft survival. Benefits persisted across clinically relevant LDKT subgroups.

**Conclusion:**

LDKT is associated with superior graft survival compared with DDKT after adjusting for confounders. Findings highlight the importance of promoting living donor programmes whilst simultaneously identifying opportunities to enhance DDKT. Future work may clarify whether factors such as reduced cold ischaemia time drive these benefits.

## Teaser

Living donor kidney transplantation (LDKT) is associated with better outcomes than deceased donation, but the causal effect remains uncertain. Using national UK registry data and inverse probability of treatment weighting, it was found that LDKT is associated with a reduced risk of graft failure, with benefits sustained both over time and across clinically relevant subgroups of living donors. These findings support efforts to expand living donor programmes.

## Introduction

End-stage renal disease (ESRD) is a life-threatening condition marked by irreversible kidney failure, typically requiring dialysis or kidney transplantation^1,2^. While dialysis is widely accessible in affluent countries, kidney transplantation is the preferred ESRD treatment in selected patients, as it offers superior quality of life and improved long-term survival outcomes^3–10^.

Kidney transplants can come from living donors (living donor kidney transplantation (LDKT)) or deceased donors (deceased donor kidney transplantation (DDKT)). Those listed for DDKT face prolonged waiting times due to limited organ availability and long cold ischaemia times (CITs) due to national organ sharing. In the UK, DDKT recipients wait approximately 2–3 years on average^11,12^. During this period, co-morbidities can develop, leading to reduced post-transplant graft survival^12–15^. In contrast, LDKT can be scheduled, with reduced waiting times and shorter CITs. Importantly, both LDKT and DDKT have better survival outcomes compared with remaining on dialysis^5,6^. In the UK, LDKT accounts for approximately 30% of kidney transplants, most donors being relatives, spouses, or friends of recipients^16^. UK living donor practices continue to change, including the use of a Kidney Sharing Scheme (KSS)^17^ and non-directed altruistic donors^18^.

Despite improvements over time, kidney transplant recipients remain at risk of graft failure and death^7,19^. Numerous recipient, donor, operative, and immunological characteristics have been linked to poorer outcomes for each type of transplantation and observational studies have suggested superior graft survival outcomes for LDKT over DDKT^7,15,19,20^. However, comprehensive contemporaneous analyses comparing LDKT and DDKT are limited, particularly in the UK^21–24^. This is important, as graft outcomes reflect national practices and organ offering policies, and findings may not be generalizable to other countries.

Furthermore, many existing studies have used standard survival analysis techniques (for example Kaplan–Meier and Cox regression) to investigate differences in graft outcomes between LDKT and DDKT^5,25–31^. However, all-cause death precludes the occurrence of graft failure and standard methods that censor deaths assume they are unrelated to donor type or graft survival, leading to overestimation of graft failure risk^32–34^. Incorporating competing risks accounts for this dependency and provides more clinically relevant estimates of graft failure risk^35,36^.

Causal inference methodologies are used when conducting an RCT is impractical or unethical. In observational data, donor and recipient characteristics may influence both choice of donor type and graft survival, introducing confounding. Inverse probability of treatment weighting (IPTW) uses these characteristics to reweight the data, so the distribution of measured confounders is comparable between LDKT and DDKT recipients, approximating the balance achieved in an RCT^37–40^. This approach reduces bias and allows for clearer understanding of how donor type influences graft survival.

The primary objective of this study was to estimate the effect of LDKT compared with DDKT on graft failure in all adult patients receiving their first, single, kidney-only transplant between 2010 and 2021. Using IPTW and treating death with a functioning graft as a competing risk, outcomes were compared had all patients received LDKT rather than DDKT, adjusting for key donor and recipient characteristics. Secondary objectives included assessing robustness of findings to missing data and verifying the validity of assumptions necessitated by causal methods. Outcomes were also compared between DDKT recipients and clinically relevant LDKT subgroups, reflective of evolving UK living donor practices or areas of clinical interest (older donors aged ≥60 years, human leucocyte antigen (HLA) mismatch level 4, and donation through the UK Kidney Sharing Scheme (KSS) or non-directed altruistic route), to determine whether any survival benefit persisted.

## Methods

### Data source and definitions

NHS Blood and Transplant manages the UK transplant registry, recording mandatory, consistent, and well-defined data for all kidney transplants performed in the UK. Data were requested for adult patients (≥18 years at time of transplant) who received their first, single, antibody-compatible, kidney-only transplant between 1 January 2010 and 31 December 2021. Donor, recipient, and immunological characteristics were collected.

Data were excluded if donor and recipient blood groups were known to be incompatible or were potentially incompatible due to missing data^41^, recipient post-transplantation outcomes were absent, or the transplantation occurred at a centre that exclusively conducted LDKT (47 patients).

The outcome for each patient was graft failure or all-cause death, whichever occurred first, and was measured in years from transplantation. Patients were censored at loss to follow-up or end of follow-up with a functioning graft.

CIT was defined by the UK transplant registry as the time between start of preservation fluid flow in the donor (or explanted kidney (live donor)) and reperfusion with the recipient’s blood. Graft failure was defined as return to chronic dialysis, graft nephrectomy, or re-transplantation, whichever occurred first. Estimated glomerular filtration rate (eGFR) was derived using the eGFRcr(AS) equation^42^ at 3, 12, and 60 months post-transplantation.

The STROBE checklist guided reporting^43^.

### Statistical methods

#### Data handling and comparisons

Data are presented as mean(s.d.) for normally distributed variables and as median (interquartile range (i.q.r.)) for non-normally distributed variables. Significant differences between donor types were tested for using *t* tests for characteristics presented as mean(s.d.), Wilcoxon rank-sum tests for characteristics presented as median (i.q.r.), and chi-squared tests for independence for categorical variables. Event times reported as 0 days were set to 0.5 days to ensure individuals entered the risk set. Post-transplantation graft function was summarized by plotting eGFR for the donor types.

#### IPTW

IPTW based on propensity scores was used to investigate the causal effect of donor type on graft survival after transplantation, treating death with a functioning graft as a competing risk. Propensity scores were estimated using a logistic regression model. All available donor, recipient, and operative characteristics that could plausibly influence both donor type and outcome were included as confounders (*Supplementary material*). Characteristics that may have been a consequence of donor type (CIT, HLA mismatch level, and waiting time) were excluded from the propensity score model as they may mediate its effect on outcomes.

Patients were weighted according to their propensity score in the outcome models, mitigating the effect of confounding between them^37–39,44^.

To assess imbalance of key variables before and after IPTW reweighting, standardized mean differences (SMDs) were calculated as the mean difference between LDKT and DDKT divided by the pooled standard deviation. A difference in SMD outside the range of −0.1 to 0.1, equivalent to a mean difference between donor types of ±0.1 s.d., indicated imbalance after reweighting^39^.

#### Competing risks

The primary outcome was graft failure, accounting for all-cause death with a functioning graft as a competing risk. Cumulative incidence functions were estimated using Aalen–Johansen estimators, weighted by IPTW for adjusted analyses. Cumulative risk of graft failure was estimated using weighted Kaplan–Meier estimators incorporating IPTW and Fine–Gray weights to adjust for the competing risk of death without graft failure. Two key causal estimates were derived: the difference in restricted mean survival time and the difference in cumulative risk of graft failure between LDKT and DDKT over 7 years post-transplantation (*Supplementary material*). HRs are not recommended in these causal methods^45^ and the proportional hazards assumption is likely violated in this setting^6^; a significant proportion of graft failures occur early post-transplantation, followed by low, constant failure rates during follow-up. Separately, these causal effects on the risk of all-cause death were estimated, with graft failure treated as a competing risk.

#### Complete case, sensitivity, and subgroup analysis

The ‘base analysis’ included only recipients with complete baseline confounders in the propensity score model. A bootstrap with 250 resamples was used to estimate the standard errors of the estimates and 95% normal confidence intervals are presented.

In sensitivity analyses, primary outcomes were re-estimated after multiple imputation of missing key confounders (*Supplementary material*). To evaluate robustness of estimates to unmeasured confounding, e-values were calculated, which quantify the minimum strength of association unmeasured confounders would need to explain the observed effect^46^. The difference in cumulative incidence of graft failure between LDKT and DDKT was re-estimated after removal of transplants performed from 2020 onward (COVID-19 period) to account for potential biases introduced by healthcare resource allocation and reduced availability of living donors^6,12,19^ and after including HLA mismatch level in the propensity model to adjust for imbalances in the quality of immunological matching between LDKT and DDKT. Subgroup analyses compared DDKT with the following LDKT subgroups: donors aged ≥60 years; transplants with HLA mismatch level 4; and LDKT performed through the UK KSS or non-directed altruistic donation route.

The validity of causal methodologies depends on key assumptions: ‘exchangeability’ (after adjusting for measured confounders, donor type is independent of potential outcomes), ‘consistency’ (observed outcomes match those under a hypothetical scenario assigning the same donor type), and ‘positivity’ (all subgroups are suitable for both LDKT and DDKT). These assumptions are discussed in the *Supplementary material* and were validated where possible. In addition, the propensity score model was assessed and patterns of missing data were explored (*Supplementary material*).

Analyses were carried out in R version 4.4.1^47^ with packages including ‘survival’^48^ (survival analysis) and ‘mice’^49^ (multiple imputation).

## Results

Data on 26 862 adults receiving their first, single, kidney-only transplant between 2010 and 2021 were analysed. There were 14 instances of known or potential blood group mismatching between recipient and donor, 177 missing recipient survival outcomes, and 47 recipients from transplant centres that performed only LDKT transplants. This resulted in 26 624 (99.1%) of the original data extract eligible for analysis (*Fig. S1*).

The base analysis comprised 10 915 (41.0%) transplantations between 2010 and 2021 with known survival outcomes and a complete set of key confounders: donor and recipient ethnicity, age, sex, and BMI, recipient blood group, cytomegalovirus (CMV) status, calculated reaction frequency, primary renal disease (PRD, *Table S1*), index of multiple deprivation (IMD), and year of transplantation. Recipient PRD, BMI, and IMD had the largest proportions of missing data.

In the base analysis, 7469 (68.4%) had a deceased donor and 3446 (31.6%) had a living donor. Comparisons between LDKT and DDKT are presented in *Table 1*. Compared with DDKT, LDKT recipients on average were younger, more likely to be CMV negative, less likely to have diabetes as PRD, had shorter CIT and waiting time, and were much more likely to be transplanted pre-emptively (before needing dialysis). Living donors were younger and more likely to be female. Recipient characteristics were broadly similar across LDKT subgroups (*Table S2*).

**Table 1.** Donor, recipient, and transplant characteristics by donor type for transplants used in the ‘base case’ analysis

Characteristic	DDKT (*n* = 7469)	LDKT (*n* = 3446)	Overall (*n* = 10 915)	*P*
**Donor sex**				<0.001
Male	4233	1552	5785	
Female	3236	1894	5130	
Donor height (cm), mean(s.d.)	171.2(9.7)	169.5(9.6)	170.6(9.7)	<0.001
Donor weight (kg), mean(s.d.)	78.7(15.4)	76.1(13.7)	77.9(14.9)	<0.001
Donor BMI (kg/m^2^), mean(s.d.)	26.8(4.8)	26.4(3.6)	26.7(4.4)	<0.001
Donor age (years), mean(s.d.)	52.4(14.0)	49.1(12.3)	51.3(13.6)	<0.001
**Donor ethnicity**				<0.001
White	7037 (94.2)	3046 (88.4)	10 083 (92.4)	
Asian	201 (2.7)	252 (7.3)	453 (4.2)	
Black	92 (1.2)	90 (2.6)	182 (1.7)	
Other	139 (1.9)	58 (1.7)	197 (1.8)	
**Donor diabetes**	559 (7.6)	NA	NA	NA
Missing	72	NA	NA	
**Donor cardiac disease**	874 (12.0)	NA	NA	NA
Missing	161	NA	NA	
**Donor creatinine at retrieval (μmol/l), median (i.q.r.)**	88.0 (75.0–112.0)	53.0 (45.0–60.0)	67.0 (52.0–89.0)	<0.001
Missing	3781	NA	NA	
**Donor urine output within previous 24 h (ml), median (i.q.r.)**	2430.0 (1587.0–3540.0)	NA	NA	NA
Missing	2577	NA	NA	
**Donor IMD quintile**				<0.001
1 (least deprived)	678 (18.5)	687 (21.8)	1365 (20.0)	
2	708 (19.3)	656 (20.8)	1364 (20.0)	
3	736 (20.0)	628 (19.9)	1364 (20.0)	
4	750 (20.4)	614 (19.5)	1364 (20.0)	
5 (most deprived)	800 (21.8)	564 (17.9)	1364 (20.0)	
Missing	3797	297	4094	
**Recipient sex**				0.287
Male	4719	2140	6859	
Female	2750	1306	4056	
Recipient height (cm), mean(s.d.)	169.9(10.3)	171.1(10.4)	170.3(10.3)	<0.001
Recipient weight (kg), mean(s.d.)	78.8(16.6)	77.9(16.7)	78.5(16.6)	0.004
Recipient BMI (kg/m^2^), mean(s.d.)	27.2(4.7)	26.5(4.6)	27.0(4.7)	<0.001
Recipient age (years), mean(s.d.)	53.3(13.3)	46.6(14.5)	51.2(14.0)	<0.001
**Recipient ethnicity**				<0.001
White	5233 (70.1)	2950 (85.6)	8183 (75.0)	
Asian	1336 (17.9)	312 (9.1)	1648 (15.1)	
Black	727 (9.7)	122 (3.5)	849 (7.8)	
Other	173 (2.3)	62 (1.8)	235 (2.2)	
**Recipient CMV**				<0.001
Negative	3293 (44.1)	1851 (53.7)	5144 (47.1)	
Positive	4176 (55.9)	1595 (46.3)	5771 (52.9)	
**Recipient blood group**				<0.001
A	2947 (39.5)	1480 (42.9)	4427 (40.6)	
AB	391 (5.2)	130 (3.8)	521 (4.8)	
B	923 (12.4)	405 (11.8)	1328 (12.2)	
O	3208 (43.0)	1431 (41.5)	4639 (42.5)	
Recipient high anti-HLA sensitization	396 (5.3)	140 (4.1)	536 (4.9)	0.006
**Recipient PRD**				<0.001
Other	4347 (58.2)	2244 (65.1)	6591 (60.4)	
Cystic kidney disease	1368 (18.3)	639 (18.5)	2007 (18.4)	
Diabetes	1286 (17.2)	348 (10.1)	1634 (15.0)	
Glomerulonephritis	468 (6.3)	215 (6.2)	683 (6.3)	
**Recipient IMD quintile**				<0.001
1 (least deprived)	1369 (18.3)	814 (23.6)	2183 (20.0)	
2	1358 (18.2)	825 (23.9)	2183 (20.0)	
3	1476 (19.8)	707 (20.5)	2183 (20.0)	
4	1575 (21.1)	608 (17.6)	2183 (20.0)	
5 (most deprived)	1691 (22.6)	492 (14.3)	2183 (20.0)	
**HLA mismatch level**				<0.001
1	574 (7.7)	316 (9.3)	890 (8.2)	
2	2177 (29.1)	522 (15.3)	2699 (24.8)	
3	3810 (51.0)	1584 (46.4)	5394 (49.6)	
4	908 (12.2)	992 (29.1)	1900 (17.5)	
Missing	0	32	32	
**Transplant year**				<0.001
2010–2012	1416 (19.0)	718 (20.8)	2134 (19.6)	
2013–2015	1764 (23.6)	913 (26.5)	2677 (24.5)	
2016–2018	2353 (31.5)	1051 (30.5)	3404 (31.2)	
2019–2021	1936 (25.9)	764 (22.2)	2700 (24.7)	
**Cold ischaemia time (h), median (i.q.r.)**	13.3 (10.3–16.8)	3.5 (2.5–4.4)	10.6 (4.7–15.1)	<0.001
Missing	34	145	179	
**Waiting time (years), median (i.q.r.)**	2.1 (1.0–3.5)	0.7 (0.3–1.5)	1.7 (0.7–3.2)	<0.001
Missing	17	1184	1201	
**Dialysis status at transplant**				<0.001
Not on dialysis	1227 (16.4)	1411 (40.9)	2638 (24.2)	
Haemodialysis	4660 (62.4)	1414 (41.0)	6074 (55.7)	
Peritoneal	1581 (21.2)	621 (18.0)	2202 (20.2)	
Missing	1	0	1	
**Outcome at end of follow-up period**				<0.001
Alive with a functioning graft	5236 (70.1)	2841 (82.4)	8077 (74.0)	
Graft failure	1115 (14.9)	319 (9.3)	1434 (13.1)	
Death with a functioning graft	1118 (15.0)	286 (8.3)	1404 (12.9)	
**Recipient creatinine at 3 months (μmol/l), median (i.q.r.)**	140.0 (111.0–180.0)	123.0 (102.0–147.0)	134.0 (107.0–168.0)	<0.001
Missing	981	312	1293	
**Recipient creatinine at 12 months (μmol/l), median (i.q.r.)**	131.0 (105.0–168.0)	118.0 (98.0–141.0)	126.0 (102.0–158.0)	<0.001
Missing	1128	346	1474	
**Recipient creatinine at 60 months (μmol/l), median (i.q.r.)**	130.0 (103.0–172.0)	119.0 (97.5–148.0)	125.0 (101.0–163.0)	<0.001
Missing	3738	1370	5108	

Values are *n* or *n* (%) unless otherwise indicated. HLA mismatch levels are defined within the UK transplant registry as follows: ‘1’ indicates no mismatches; ‘2’ represents zero mismatches at the DR locus and zero or one at the B locus; ‘3’ includes cases with either zero mismatches at the DR locus and two at the B locus, or one mismatch at the DR locus and zero or one at the B locus; and ‘4’ includes cases with two mismatches at the DR locus, or one at the DR locus and two at the B locus. High anti-HLA sensitization is defined as a recipient calculated reaction frequency >85%. DDKT, deceased donor kidney transplantation; LDKT, living donor kidney transplantation; NA, not available; i.q.r., interquartile range; IMD, index of multiple deprivation; CMV, cytomegalovirus; HLA, human leucocyte antigen; PRD, primary renal disease.

**Fig. 1:**
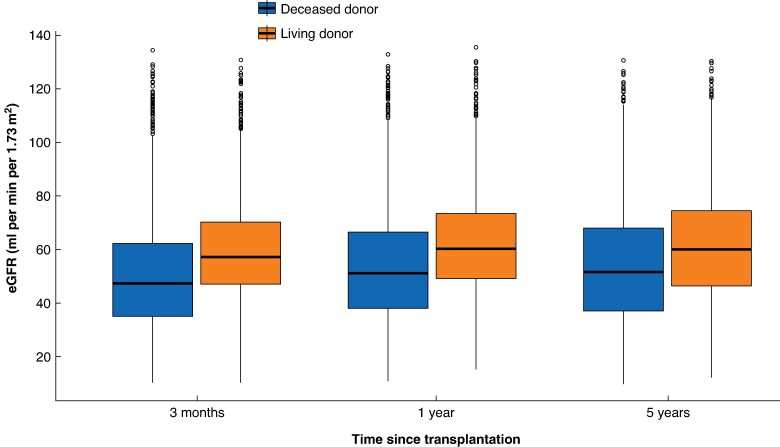
eGFR at 3 months, 1 year, and 5 years post-transplantation stratified by donor type Boxes show the median and i.q.r. values, and whiskers extend to 1.5 × i.q.r. Profiles are shown for recipients in the ‘base case’ analysis. At 3 months there were 6415 (85.9%) non-missing eGFR measurements for DDKT recipients and 3123 (90.6%) non-missing eGFR measurements for LDKT recipients, at 1 year there were 6277 (84.0%) non-missing eGFR measurements for DDKT recipients and 3085 (89.5%) non-missing eGFR measurements for LDKT recipients, and at 5 years there were 3675 (49.2%) non-missing eGFR measurements for DDKT recipients and 2054 (59.6%) non-missing eGFR measurements for LDKT recipients. eGFR, estimated glomerular filtration rate; i.q.r., interquartile range; DDKT, deceased donor kidney transplantation; LDKT, living donor kidney transplantation.

Graft failure occurred in 1115 (14.9%) DDKT recipients and 319 (9.3%) LDKT recipients. In exploratory analysis, black recipients, those from most-deprived areas, those on haemodialysis before transplantation, and those with HLA mismatch level ≥2 had the largest univariable HRs associated with graft failure (*Table S3*). Death without graft failure occurred in 1118 (15.0%) DDKT recipients and 286 (8.3%) LDKT recipients. Post-transplantation, the median eGFR was approximately ten units higher in LDKT recipients compared with DDKT recipients (*Fig. 1*).

**Fig. 2:**
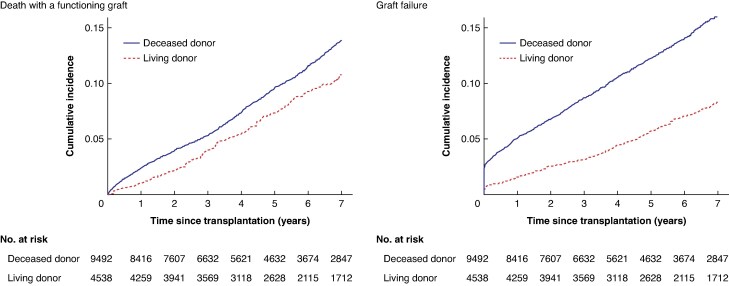
Cumulative incidence function for the competing risks of graft failure and all-cause death with a functioning graft after IPTW based on the same ‘base analysis’ The two panels are additive, with their combination reflecting the total incidence of events throughout follow-up. IPTW, inverse probability of treatment weighting.

**Fig. 3:**
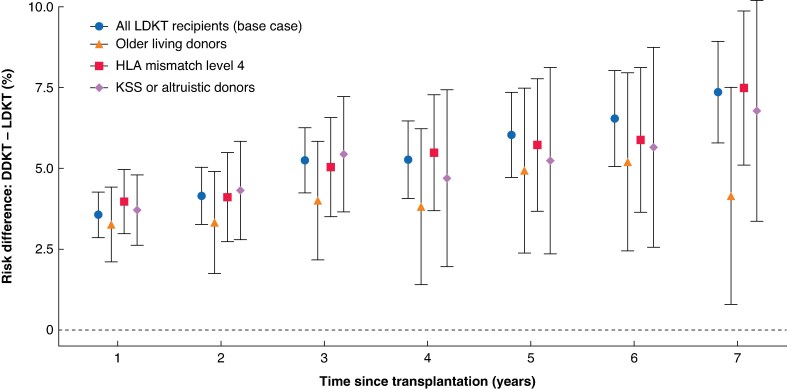
Mean estimates (points) and 95% confidence intervals (error bars) for the absolute risk difference in graft failure (DDKT − LDKT) 1–7 years post-transplantation Confidence intervals were calculated as the mean value ± 1.96 × standard deviation across 250 bootstrap samples. Analyses included the base case and key clinically relevant subgroups of living donors. LDKT, living donor kidney transplantation; DDKT, deceased donor kidney transplantation; HLA, human leucocyte antigen; KSS, Kidney Sharing Scheme.

After adjustment, incidence of graft failure at 5 years post-transplantation was 5.61% (95% c.i. 4.60% to 6.61%) for LDKT recipients and 11.63% (95% c.i. 10.82% to 12.44%) for DDKT recipients, a benefit of 6.03% (95% c.i. 4.71% to 7.35%) (*Fig. 2*). LDKT conferred an additional 0.36 (95% c.i. 0.29 to 0.43) years without graft failure over 7 years. The benefit for graft failure incidence increased from 3.56% (95% c.i. 2.86% to 4.26%) at 1 year to 7.36% (95% c.i. 5.78% to 8.94%) at 7 years post-transplantation (*Fig. 3*). Subgroup analyses (*Fig. 3* and *Table S4*) showed similar benefit for kidneys from older living donors (5-year risk difference 4.93%), for level 4 HLA-matched grafts (5-year risk difference 5.72%), and LDKT via the UK KSS or non-directed altruistic donation route (5-year risk difference 5.24%).

Incidence of death with a functioning graft after adjustment at 5 years post-transplantation was 7.11% (95% c.i. 5.76% to 8.45%) for LDKT and 9.60% (95% c.i. 8.93% to 10.26%) for DDKT, a small benefit of 2.49% (95% c.i. 1.03% to 3.94%). The benefit for all-cause death was small, but significant, throughout follow-up (*Table S5*).

**Fig. 4:**
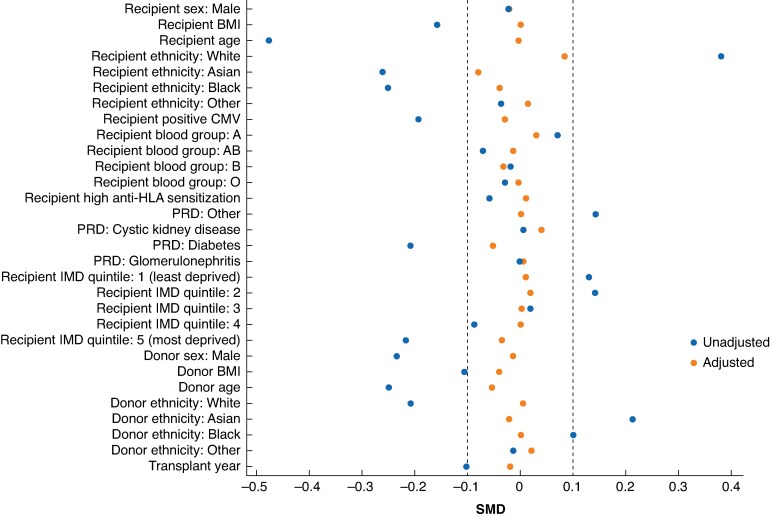
SMDs before (‘unadjusted’) and after (‘adjusted’) IPTW Confounders on the *y*-axis were present in the propensity score model and the balance between living and deceased donors was measured using SMDs. The area between the vertical dashed lines at ±0.1 indicates a reasonable balance between the donor groups. SMDs, standardized mean differences; IPTW, inverse probability of treatment weighting; CMV, cytomegalovirus; HLA, human leucocyte antigen; PRD, primary renal disease; IMD, index of multiple deprivation.

Before adjustment, donor and recipient age and ethnicity showed noticeable imbalance across donor types (*Fig. 4*). After applying IPTW reweighting, these differences were substantially reduced across all confounders. Adjusted differences were within the ±0.1 s.d. range, indicating successful mitigation of bias for these known confounders. The propensity score model fitted the data well (*supplementary methods S1* and *Figs S2*, *S3*).

### Sensitivity analyses

There were high rates of missing data for some variables. However, multiple imputation of missing data for key confounders using data from 26 624 transplants did not alter estimates (*Tables S4*, *S5*). The median e-value for the risk difference at 1 year was 6.99 (i.q.r. 6.08–7.95), indicating that unmeasured confounders would require an associated HR of approximately 7 to explain the observed effect. Corresponding median e-values were 3.57 (i.q.r. 3.33–3.88) and 3.19 (i.q.r. 2.98–3.40) at 5 years and 7 years respectively. Exclusion of 1596 transplantations performed during the COVID-19 period (2020 onwards) slightly increased the estimated 1-year risk difference between LDKT and DDKT to 3.95% (95% c.i. 3.24% to 4.67%), suggesting that inclusion of COVID-19-era transplants slightly attenuated the observed benefit of LDKT; incorporation of HLA mismatch level into the propensity score model did not alter risk difference estimates over follow-up (*Tables S4*, *S5*).

## Discussion

LDKT was associated with a significant reduction in graft failure risk compared with DDKT in a contemporary UK cohort. After adjusting for key donor and recipient characteristics, and treating death with a functioning graft as a competing risk, 5-year graft failure risk was substantially lower for LDKT *versus* DDKT and results suggested that the benefit increased over time post-transplantation. The benefit of living donation persisted across sensitivity analyses and was observed consistently in clinically relevant LDKT subgroups, including older donors, poorly matched grafts, and donations via the UK KSS or non-directed altruistic route.

Analyses treated shorter CITs and waiting times and higher pre-emptive transplantation rates as part of the overall ‘treatment package’ of living donation. The approach reflects real-world practice and many of the rationales for promoting living donor programmes. Mediation analyses could quantify the extent to which observed benefits are attributable to (for example) the reduction in CIT^17,50–52^. This approach could provide specific mechanistic insights, but assumes a hypothetical scenario where CIT can be made comparable across donor types. This might occur in the future, such as after expansion of the UK KSS^17^. Similar issues also apply to the markedly different rates of pre-emptive transplantation between the two recipient groups.

The findings build on prior research that reported both superior graft survival for LDKT^25,26^ and comparable patient survival rates between LDKT and DDKT^27,28^, but which was limited by smaller, single-centre cohorts, standard survival methods not accounting for competing risks, and potential confounding. By using IPTW and competing risks models, robust estimates of the causal effect of donor type are estimated. Recipient, donor, and operative factors are known to influence graft outcomes. Recipient co-morbidities are associated with increased risks of graft failure and mortality across donor types^53^. Outcomes have improved over time, particularly among diabetic, older, and black recipients^7^. Recipients of kidneys from older deceased donors have poorer outcomes than recipients of kidneys from standard criteria deceased donors^54^. While LDKT is associated with better early post-transplant quality of life^3^, longer-term trajectories appear similar between donor types, with both preferable to remaining on the transplant waiting list^4^.

A UK study previously reported that kidneys from older (≥60 years) living donors were associated with lower graft failure risk compared with younger standard criteria deceased donors, supporting expansion of the living donor pool^6^. The present analyses complement this research by applying causal methodologies and demonstrate that the benefit of LDKT persists across key clinically relevant subgroups, including older living donors, grafts with poor HLA matching, and transplants via the UK KSS or non-directed altruistic donation route.

This study used comprehensive, high-quality, standardized national registry data, with a large cohort and long follow-up spanning multiple transplantation eras, strengthening generalizability to UK adult transplant candidates and similar populations and services. Use of causal methods and competing risks models, with multiple sensitivity analyses (multiple imputation, adjustment for HLA mismatch level, and exclusion of COVID-19-era transplants), provides robust estimates of the effect of donor type. Subgroup analyses added insight regarding a consistent benefit across key LDKT subgroups, reflecting evolving UK LDKT.

However, in the present study, the absence of comprehensive data on recipient co-morbidities, dialysis duration, and ESRD duration limited the ability to adjust for recipient complexity. Although the propensity score model achieved balance of observed confounders, unmeasured confounders likely remain, including genetic, molecular, and other biomarkers that could lead to personalized donor selection. Estimated e-values suggest that unmeasured confounders would require a ‘joint’ HR of approximately 7 at 1 year and approximately 3 at 3 years to explain the observed benefit; robustness to unmeasured confounding may diminish over follow-up. Additionally, ABO- and HLA-incompatible LDKT were excluded from analyses, though it is recognized that these represent important types of LDKT, the outcomes of which require ongoing study^55–59^. Finally, inclusion of COVID-19-era transplants may have introduced biases, as the pandemic reduced the number of LDKT performed and redirected healthcare resources^6,12,19^. The present study focused on graft failure and survival outcomes post-transplantation; studies of recipients’ quality of life, performance status, and kidney function would offer further insight into the relative benefits of donor types.

Despite its benefits, access to LDKT remains inequitable. LDKT recipients tend to be younger and less likely to have diabetes than DDKT recipients, with variation across countries^60^. Socio-economic deprivation is a significant barrier; individuals from lower socio-economic groups have reduced opportunities for LDKT, reportedly driven by factors such as low patient activation, limited knowledge of LDKT^61,62^, and limited health literacy^63^. Targeted interventions may reduce inequities^64^. In the UK, significant disparities in LDKT uptake exist according to age, ethnicity, socio-economic status, and geography^65^. Individuals from areas of high socio-economic deprivation reported low confidence and skills in managing their health, further limiting access^66^.

These findings, alongside previous research comparing donor types, provide valuable insights for decision makers and a useful resource for dialogue between kidney transplant candidates and their care teams. Although the findings of the present study underscore the value of living kidney donor programmes, it is acknowledged that these programmes rely on the willingness of healthy individuals to undergo major surgery, raising important ethical considerations. It is essential that patients, clinicians, and the general public are better informed about both living and deceased donation and that countries invest appropriately in both, to ensure equitable access to life-saving and life-enhancing organ transplantation.

## Supplementary Material

znaf162_Supplementary_Data

## Data Availability

The data used in this study are publicly available, but approval is required for access. Data were obtained from NHS Blood and Transplant and can be requested through their application process (https://www.odt.nhs.uk/statistics-and-reports/access-data/).
