# Supplementary material for: Comparison of outcomes after living and deceased donor kidney transplantation: UK national cohort study
Source: Br J Surg. 2025 Aug 19;112(8):znaf162. doi: 10.1093/bjs/znaf162 (PMC12362070; doi:10.1093/bjs/znaf162)
Supplement: znaf162_Supplementary_Data [file znaf162_supplementary_data.docx]

**Supplementary Material to “Comparison of outcomes after living and deceased donor kidney transplantation: A United Kingdom national cohort study”**

James Murray^a^, Annabel Luke^a^, David Wallace^a,b^*, Chris Callaghan^c^*, Linda D. Sharples^a^*

a: Department of Medical Statistics, London School of Hygiene and Tropical Medicine, Keppel Street, London, WC1E 7HT, UK

b: Department of HPB and Liver Transplant, The Royal Free Hospital, NHS Foundation Trust, Pond Street, London, NW3 2Q, UK

c: Department of Nephrology and Transplantation, Guy’s Hospital, Guys’ and St. Thomas’ NHS Foundation Trust, Great Maze Pond, London, SE1 9RT, UK

*: Senior authorship is shared equally by DW, CC (clinical) and LDS (methodology)

**Corresponding Author** Professor Linda Sharples, London School of Hygiene and Tropical Medicine, Keppel Street, London, WC1E 7HT, UK.

| **Supplementary Materials - Index**  [Supplementary Methods 2](#_Toc200101950)  [S1 Propensity score model and inverse probability of treatment weights 2](#_Toc200101951)  [S2 Competing risks analysis 3](#_Toc200101952)  [S3 Assumptions for causal analysis 4](#_Toc200101953)  [S4 Missing data 4](#_Toc200101954)  [Supplementary Tables and Figures 7](#_Toc200101955)  [Supplementary Table S1: Recipient primary renal disease 7](#_Toc200101956)  [Supplementary Table S2: Characteristics for LDKT subgroups 9](#_Toc200101957)  [Supplementary Table S3: Univariable associations with event times 12](#_Toc200101958)  [Supplementary Table S4: Incidence and risk difference of graft failure 13](#_Toc200101959)  [Supplementary Table S5: Incidence and risk difference of death with functioning graft 14](#_Toc200101960)  [Supplementary Table S6: Estimated coefficients from propensity score model 15](#_Toc200101961)  [Supplementary Table S7: Associations between missing data and competing risks 16](#_Toc200101962)  [Supplementary Figure S1: Flowchart of data exclusions and missing data 17](#_Toc200101963)  [Supplementary Figure S2: Propensity score model – deviance residuals 18](#_Toc200101964)  [Supplementary Figure S3: Propensity score model – distribution of predicted probabilities 18](#_Toc200101965)  [Supplementary Figure S4: Patterns of missing data 19](#_Toc200101966)  [References 22](#_Toc200101967) |  |
| --- | --- |
|  |  |
|  |  |

#

# Supplementary Methods

## S1 Propensity score model and inverse probability of treatment weights

To address potential confounding in the comparison of living donor kidney transplantation (LDKT) and deceased donor kidney transplantation (DDKT), a propensity score model was constructed. Propensity scores estimate the probability of receiving a transplant from a living donor ($Y_{i}=1$) versus a deceased donor ($Y_{i}=0$) for patient $i$ based on observed recipient, donor, and operative characteristics. These scores are used to calculate inverse probability of treatment weights (IPTWs), which balance the distribution of confounders across the two donor groups thereby mitigating biases in estimation of average treatment effects.

The propensity score model is the logistic regression model

$$\text{logit}\left( E_{i}\left[ Y_{i}=1 | R_{i},D_{i},O_{i} \right] \right)=\beta_{0}+R_{i}^{\top}\beta_{R}+D_{i}^{\top}\beta_{D}+O_{i}^{\top}\beta_{O},$$

where $R_{i},D_{i},$ and $O_{i}$ denote vectors of recipient, donor, and operative characteristics, respectively, with $\beta_{R},\beta_{D},$ and $\beta_{O}$ the corresponding vector of coefficients to be estimated.

The contribution of **recipient** characteristics to the linear predictor is

$$R_{i}^{\top}\beta_{R}=\beta_{R1}I\left( \text{Sex}_{i}=\text{Male} \right)+\beta_{R2}\left( \text{BMI}_{i}-\overline{\text{BMI}} \right)+\beta_{R3}\left( \text{Age}_{i}-\overline{\text{Age}} \right)+\beta_{R4}I\left( \text{Ethnicity}_{i}=\text{Asian} \right)+\beta_{R5}I\left( \text{Ethnicity}_{i}=\text{Black} \right)+\beta_{R6}I\left( \text{Ethnicity}_{i}=\text{Other} \right)+\beta_{R7}I\left( \text{CMV}_{i}=\text{Positive} \right)+\beta_{R8}I\left( \text{BG}_{i}=\text{AB} \right)+\beta_{R9}I\left( \text{BG}_{i}=\text{B} \right)+\beta_{R10}I\left( \text{BG}_{i}=\text{O} \right)+\beta_{R11}I\left( \text{cRF}_{i}>85\% \right)+\beta_{R12}I\left( \text{PRD}_{i}=\text{Diabetes} \right)+\beta_{R13}I\left( \text{PRD}_{i}=\text{Cystic kidney disease} \right)+\beta_{R14}I\left( \text{PRD}_{i}=\text{Glomerulonephritis} \right)+\beta_{R15}I\left( \text{IMD}_{i}=2 \right)+\beta_{R16}I\left( \text{IMD}_{i}=3 \right)+\beta_{R17}I\left( \text{IMD}_{i}=4 \right)+\beta_{R18}I\left( \text{IMD}_{i}=5 \right)$$

Where $I\left( \cdot\right)$ denotes the indicator function, taking value one if its argument is true and zero otherwise. In the above, ‘cRF’ denotes calculated reaction frequency and determines whether the recipient had high anti-HLA sensitisation; blood group (‘BG’) and primary renal disease (‘PRD’) have reference categories ‘A’ and ‘other’, respectively. The reference groups for sex, ethnicity, cytomegalovirus (‘CMV’), and index of multiple deprivation quintile (‘IMD’) were female, white, negative, and 1 (least deprived), respectively. Continuous covariates were mean-centered, overbars denoting mean values.

Similarly, the **donor** characteristics in the propensity score model were

$$D_{i}^{\top}\beta_{D}=\beta_{D1}I\left( \text{Sex}_{i}=\text{Male} \right)+\beta_{D2}\left( \text{BMI}_{i}-\overline{\text{BMI}} \right)+\beta_{D3}\left( \text{Age}_{i}-\overline{\text{Age}} \right)+\beta_{D4}I\left( \text{Ethnicity}_{i}=\text{Asian} \right)+\beta_{D5}I\left( \text{Ethnicity}_{i}=\text{Black} \right)+\beta_{D6}I\left( \text{Ethnicity}_{i}=\text{Other} \right).$$

Finally, the ‘**operative**’ characteristics of the transplantation were

$$O_{i}^{\top}\beta_{O}=\beta_{O1}\left( \text{Transplant }\text{year}_{i}-2010 \right).$$

From the propensity score model, we obtain the predicted probabilities of receiving a living donor, conditional on the listed identified confounders

$$P_{i}=\hat{Pr}\left( Y_{i}=1 | R_{i},D_{i},O_{i} \right).$$

The IPTWs ($W_{i}$) are subsequently calculated as

$$W_{i}=\left\{ \begin{aligned} \frac{1}{P_{i}} \text{if} Y_{i}=1 \\ \frac{1}{1-P_{i}} \text{if} Y_{i}=0. \end{aligned} \right.$$

These weights are applied to balance confounders across living and deceased donor groups. Diagnostic plots from the propensity score model for the “base case” are presented in Figures S2 and S3, alongside estimated regression coefficients in Table S5. The model's deviance residuals in Figure S2 appear approximately normal with no obvious outliers. Estimated regression coefficients are presented in Table S6 for the base case.

## S2 Competing risks analysis

The Fine and Gray sub-distribution hazard model is used to estimate the cumulative incidence function (CIF) for graft failure whilst accounting for death with a functioning graft as a competing risk. This models the probability of graft failure over follow-up whilst considering dependency between competing events (e.g., death precludes graft failure). This provides causal estimates for the effect of receiving LDKT compared to DDKT. The CIF for graft failure at time $t$, denoted $\text{CIF}_{\text{LDKT}}\left( t \right)$ and $\text{CIF}_{\text{DDKT}}\left( t \right)$ for LDKT and DDKT recipients, respectively, represents the probability of experiencing graft failure by time $t$, in the presence of competing risks.

The weights used in analyses were the product of the IPTWs (Section S1) and the weights obtained from the Fine and Gray procedure, which are dynamically modified throughout follow-up as the risk sets change due to occurrence of death without graft failure. The combined (product of) weights were applied to a weighted Kaplan-Meier estimator for the cumulative risk of graft failure.

The observed event time for subject $i$ is $T_{i}=\min\left( G_{i},D_{i},C_{i} \right)$ where $G_{i}$ the time of graft failure, $D_{i}$ death with functioning graft, and $C_{i}$ censoring. For main analysis we introduce event indicator $\delta_{i}=1$ if $T_{i}=G_{i}$ and $\delta_{i}=0$ otherwise. Two interpretable causal estimates were derived for $t=1,\ldots,7$ years post-transplantation.

1. The difference in cumulative risk of graft failure between donor types at time $t$.

$$\Delta\text{Risk}\left( t \right)=\text{CIF}_{\text{LDKT}}\left( t \right)-\text{CIF}_{\text{DDKT}}\left( t \right),$$

$$\Delta\text{Risk}\left( t \right)=\Pr\left( T_{i}\leq t,\delta_{i}=1 | \text{LDKT} \right)-\Pr\left( T_{i}\leq t,\delta_{i}=1 | \text{DDKT} \right),$$

which provides a direct comparison of the probabilities of experiencing graft failure by time $t$ between LDKT and DDKT.
2. The difference in restricted mean survival time (RMST), representing the expected amount of time free of graft failure, up to time $t$. This is defined as

$$\Delta\text{RMST}\left( t \right)=\int_{0}^{t} S_{\text{LDKT}}\left( u \right)-S_{\text{DDKT}}\left( u \right) du$$

where $S_{\text{LDKT}}\left( u \right)=1-\text{CIF}_{\text{LDKT}}\left( u \right)$ and $S_{\text{DDKT}}\left( u \right)=1-\text{CIF}_{\text{DDKT}}\left( u \right)$ are the donor-specific survival probabilities of being free from graft failure up to time $u$. This causal estimate represents the average additional time free of graft failure up to time $t$ for LDKT compared to DDKT.

## S3 Assumptions for causal analysis

Causal analyses are dependent on several key assumptions. We consider each in turn and discuss their validity in the presented analyses.

*Consistency* assumes that, for each recipient, the observed outcome under their received donor type is the same as it would have been under a hypothetical scenario assigning the same donor type. In this context, LDKT and DDKT must be well-defined across all patients, with no ambiguity in their administration. Transplantation procedures (regardless of donor type) in the UK follow standardised protocols, ensuring consistency in treatment. Therefore, we are confident there were no violations of this assumption due to systematic differences in the standard of care post-transplantation.

*Conditional exchangeability* assumes that, given measured confounders, actual donor type is independent of potential outcomes. That is, the measured covariates are sufficient to explain non-causal associations between donor type and graft failure. The propensity score model in Section S1 provides a method of reweighting patients' contributions to parameter estimation, so that comparisons between donor types are balanced for key recipient and donor characteristics. However, potentially important characteristics such as genealogical, molecular, or biological markers of end stage renal disease severity prior to transplantation which could lead to personalized donor selection were not available and could confer unmeasured confounding. The $e$-values in the main paper suggest that any unmeasured confounders would need strong associations with graft failure to fully explain the observed effects. While we are confident findings are robust to large individual unmeasured confounders, we acknowledge that combinations of more moderate confounders could introduce bias, particularly later in follow-up.

*Positivity* assumes that it is possible for each patient to have received both LDKT and DDKT:

$$0<P_{i}<1 \forall i.$$

In our analyses, all patients received transplantation, and no subpopulations lacked access to living donors. Specialist transplantation centres that exclusively conducted LDKT were excluded to ensure this assumption was met. In theory, any patient could have received *either* DDKT or LDKT if a compatible donor became available. In the base analysis, $P_{i}$ ranged from 0.022 to 0.937, and Figure S3 demonstrates reasonable overlap in the distribution of propensity scores between LDKT and DDKT recipients. With these in mind we were confident that the positivity assumption was reasonable.

## S4 Missing data

To investigate patterns of missing data and verify assumptions necessary for multiple imputation, we conducted several analyses. Using the 26 624 individuals eligible for analyses, we created binary indicators for each confounder in the propensity score model (Section S1) to flag missingness alongside a *composite* indicator denoting whether complete data were available for each transplant. Figure S4 illustrates patterns of missing data and their frequency for the outcome and key covariates the propensity score model.

Log-rank tests were used to assess differences in the cumulative incidence of the competing risks, stratified by missingness indicators for individual covariates, as well as by the composite indicator for one or more missing covariates. This aimed to ascertain whether missingness was associated with patient outcomes. Results are presented in Table S7. Generally, variables with higher proportions of missingness, and overall completeness of data, are associated with differences in graft failure incidence, and recipient ethnicity is associated with both incidence of graft failure and death with functioning graft. This suggests the data are unlikely to be missing completely at random, as missingness appears to be associated with fully observed outcomes.

In conventional multiple imputation with $M$ imputed datasets, estimating within-imputation variance would require performing $B$ bootstrap resamples within *each* imputed dataset due to the absence of a variance estimator for the causal estimands. This results in significant computational burden. Bartlett & Hughes (2020) present and evaluate alternative approaches. Of these, we employ the ‘*Boot MI percentile*’ approach, which uses $M=1$ imputation within *each* of the $B$ bootstrap samples of the incomplete data. This strategy yields $B$ estimates of the average treatment effect, allowing empirical 95% confidence intervals to be derived directly from the distribution of the estimates. This method then significantly improves computational efficiency as it reduces the need for repeated imputations within each bootstrap sample.

For each of the $B=250$ bootstrap samples, the imputation model incorporates key confounders from the propensity score model (Section S1), along with the patient's outcome at the end of follow-up and the logarithm of their observed final follow-up time (i.e., censoring or occurrence of either outcome). Imputations were performed separately for recipients of LDKT and DDKT.

# Supplementary Tables and Figures

## Supplementary Table S1: Recipient primary renal disease

Prevalence of primary renal disease grouped as ‘cystic kidney disease’, ‘diabetes’, ‘glomerulonephritis’, or ‘other’ for analyses, split by donor type. Primary renal disease is arranged by these analysis groups and by overall prevalence within-group. NHSBT: NHS Blood & Transplant.

| Primary renal disease reported by NHSBT n (%) | DDKT  N = 7469 | LDKT  N = 3446 | Overall  N = 10 915 |
| --- | --- | --- | --- |
| **Cystic Kidney Disease** | | | |
| Polycystic kidneys, adult type (dominant type) | 1293 (17.31%) | 605 (17.56%) | 1898 (17.39%) |
| Cystic kidney disease - type unspecified | 43 ( 0.58%) | 15 ( 0.44%) | 58 ( 0.53%) |
| Medullary cystic disease, including nephronophthisis | 16 ( 0.21%) | 10 ( 0.29%) | 26 ( 0.24%) |
| Cystic kidney disease - other specified type | 12 ( 0.16%) | 3 ( 0.09%) | 15 ( 0.14%) |
| Polycystic kidneys, infantile (recessive type) | 4 ( 0.05%) | 6 ( 0.17%) | 10 ( 0.09%) |
| **Diabetes** | | | |
| Diabetes - non-insulin dependent | 922 (12.34%) | 163 (4.73%) | 1085 (9.94%) |
| Diabetes - insulin dependent | 364 ( 4.87%) | 185 (5.37%) | 549 (5.03%) |
| **Glomerulonephritis** | | | |
| Glomerulonephritis, histologically examined | 239 (3.20%) | 87 (2.52%) | 326 (2.99%) |
| Glomerulonephritis, histologically not examined | 122 (1.63%) | 76 (2.21%) | 198 (1.81%) |
| Membrano - proliferative glomerulonephritis | 83 (1.11%) | 34 (0.99%) | 117 (1.07%) |
| Rapidly progressive GN without systemic disease | 24 (0.32%) | 15 (0.44%) | 39 (0.36%) |
| Cryoglobulinemic glomerulonephritis | 0 (0.00%) | 3 (0.09%) | 3 (0.03%) |
| **Other** | | | |
| IgA nephropathy | 912 (12.21%) | 628 (18.22%) | 1540 (14.11%) |
| Other identified renal disorders | 871 (11.66%) | 430 (12.48%) | 1301 (11.92%) |
| Renal vascular disease - hypertension | 579 ( 7.75%) | 220 ( 6.38%) | 799 ( 7.32%) |
| Focal segmental glomerulosclerosis with nephrotic syndrome in adults | 278 ( 3.72%) | 142 ( 4.12%) | 420 ( 3.85%) |
| Pyelonephritis/Interstitial nephritis due to V-U reflux without obstruction | 174 ( 2.33%) | 114 ( 3.31%) | 288 ( 2.64%) |
| Lupus erythematosus | 128 ( 1.71%) | 56 ( 1.63%) | 184 ( 1.69%) |
| Renal vascular disease - malignant hypertension | 152 ( 2.04%) | 28 ( 0.81%) | 180 ( 1.65%) |
| Pyelonephritis/Interstitial nephritis - cause not specified | 117 ( 1.57%) | 60 ( 1.74%) | 177 ( 1.62%) |
| Membranous nephropathy | 129 ( 1.73%) | 43 ( 1.25%) | 172 ( 1.58%) |
| Pyelonephritis/Interstitial nephritis due to con obs uropathy with/without V-U reflux | 68 ( 0.91%) | 45 ( 1.31%) | 113 ( 1.04%) |
| Hereditary nephritis with nerve deafness (Alports syndrome) | 70 ( 0.94%) | 32 ( 0.93%) | 102 ( 0.93%) |
| Tubulo Interstitial Nephritis (Not Pyelonephritis) | 65 ( 0.87%) | 34 ( 0.99%) | 99 ( 0.91%) |
| Congenital renal dysplasia with or without urinary tract malformation | 51 ( 0.68%) | 43 ( 1.25%) | 94 ( 0.86%) |
| Goodpasture's Syndrome | 57 ( 0.76%) | 36 ( 1.04%) | 93 ( 0.85%) |
| Wegener's granulomatosis | 63 ( 0.84%) | 29 ( 0.84%) | 92 ( 0.84%) |
| Nephropathy caused by other specific drug | 64 ( 0.86%) | 24 ( 0.70%) | 88 ( 0.81%) |
| Pyelonephritis/Interstitial nephritis due to acquired obstructive uropathy | 63 ( 0.84%) | 24 ( 0.70%) | 87 ( 0.80%) |
| Amyloid | 47 ( 0.63%) | 20 ( 0.58%) | 67 ( 0.61%) |
| Pyelonephritis/Interstitial nephritis due to other cause | 32 ( 0.43%) | 20 ( 0.58%) | 52 ( 0.48%) |
| Renal vascular disease - type unspecified | 40 ( 0.54%) | 11 ( 0.32%) | 51 ( 0.47%) |
| Henoch-Schonlein purpura | 26 ( 0.35%) | 24 ( 0.70%) | 50 ( 0.46%) |
| Hereditary/Familial nephropathy - type unspecified | 28 ( 0.37%) | 15 ( 0.44%) | 43 ( 0.39%) |
| Congenital renal hypoplasia - type unspecified | 25 ( 0.33%) | 13 ( 0.38%) | 38 ( 0.35%) |
| Nephrocalcinosis & hypercalcaemic nephropathy | 26 ( 0.35%) | 10 ( 0.29%) | 36 ( 0.33%) |
| Haemolytic Uraemic Syndrome (inc Moschowitz Syndrome) | 17 ( 0.23%) | 18 ( 0.52%) | 35 ( 0.32%) |
| Traumatic or surgical loss of kidney | 21 ( 0.28%) | 11 ( 0.32%) | 32 ( 0.29%) |
| Hereditary nephropathy - other | 21 ( 0.28%) | 8 ( 0.23%) | 29 ( 0.27%) |
| Nephropathy due to cyclosporin A | 15 ( 0.20%) | 13 ( 0.38%) | 28 ( 0.26%) |
| Pyelonephritis/Interstitial nephritis associated with neurogenic bladder | 15 ( 0.20%) | 13 ( 0.38%) | 28 ( 0.26%) |
| Pyelonephritis/Interstitial nephritis due to urolithiasis | 17 ( 0.23%) | 11 ( 0.32%) | 28 ( 0.26%) |
| Cortical or tubular necrosis | 18 ( 0.24%) | 8 ( 0.23%) | 26 ( 0.24%) |
| Severe nephrotic syndrome with focal sclerosis | 17 ( 0.23%) | 8 ( 0.23%) | 25 ( 0.23%) |
| Ischaemic renal disease/ cholesterol embolism | 11 ( 0.15%) | 8 ( 0.23%) | 19 ( 0.17%) |
| Kidney tumour | 16 ( 0.21%) | 3 ( 0.09%) | 19 ( 0.17%) |
| Renal vascular disease - polyarteritis | 15 ( 0.20%) | 3 ( 0.09%) | 18 ( 0.16%) |
| Renal vascular disease - classified | 14 ( 0.19%) | 3 ( 0.09%) | 17 ( 0.16%) |
| Systemic sclerosis (Scleroderma) | 13 ( 0.17%) | 4 ( 0.12%) | 17 ( 0.16%) |
| Myelomatosis/Light chain deposit disease | 10 ( 0.13%) | 5 ( 0.15%) | 15 ( 0.14%) |
| Cystinosis | 8 ( 0.11%) | 6 ( 0.17%) | 14 ( 0.13%) |
| Fabry's disease | 10 ( 0.13%) | 4 ( 0.12%) | 14 ( 0.13%) |
| Multi-system disease - other | 9 ( 0.12%) | 3 ( 0.09%) | 12 ( 0.11%) |
| Tuberculosis | 12 ( 0.16%) | 0 ( 0.00%) | 12 ( 0.11%) |
| Dense deposit disease | 3 ( 0.04%) | 8 ( 0.23%) | 11 ( 0.10%) |
| Nephropathy due to analgesic drugs | 8 ( 0.11%) | 3 ( 0.09%) | 11 ( 0.10%) |
| Gout | 3 ( 0.04%) | 0 ( 0.00%) | 3 ( 0.03%) |
| Primary oxalosis | 3 ( 0.04%) | 0 ( 0.00%) | 3 ( 0.03%) |
| Syndrome of agenesis of abdominal muscles | 1 ( 0.01%) | 2 ( 0.06%) | 3 ( 0.03%) |
| Nephropathy due to cis-platinum | 2 ( 0.03%) | 0 ( 0.00%) | 2 ( 0.02%) |
| Oligomeganephronic hypoplasia | 1 ( 0.01%) | 1 ( 0.03%) | 2 ( 0.02%) |
| Segmental renal hypoplasia | 2 ( 0.03%) | 0 ( 0.00%) | 2 ( 0.02%) |

## Supplementary Table S2: Characteristics for LDKT subgroups

Split by donor type for transplantations used in the ‘base analysis’ for deceased donors (DDKT) and living donor (LDKT) subgroups. Human leukocyte antigen (HLA) mismatch levels are defined within the UK transplant registry as follows: ‘1’ indicates no mismatches; ‘2’ represents zero mismatches at the DR locus and zero or one at the B locus; ‘3’ includes cases with either zero mismatches at the DR locus and two at the B locus, or one mismatch at the DR locus and zero or one at the B locus; and ‘4’ includes cases with two mismatches at the DR locus, or one at the DR locus and two at the B locus. High anti-HLA sensitisation is defined as recipient-calculated reaction frequency exceeding 85%. KSS: Kidney Sharing Scheme; BMI: Body mass index; CMV: Cytomegalovirus; PRD: Primary renal disease; IMD: Index of multiple deprivation; SD: Standard deviation; IQR: Interquartile range; -: Data not available.

| **Characteristic** | **DDKT**  N = 7469 | **Older LDKT donors**  N = 760 | **LDKT, HLA mismatch**  **level 4**  N = 992 | **LDKT, KSS or**  **altruistic**  N = 563 |
| --- | --- | --- | --- | --- |
| **Donor sex ratio (M:F)** | 4233:3236 | 308:452 | 415:577 | 278:285 |
| **Mean (SD) donor height (cm)** | 171.2 (9.7) | 167.3 (9.2) | 169.1 (9.4) | 170.3 (9.4) |
| **Mean (SD) donor weight (kg)** | 78.7 (15.4) | 73.5 (12.2) | 75.8 (13.9) | 75.5 (13.5) |
| **Mean (SD) donor BMI (kg/cm^2)** | 26.8 (4.8) | 26.2 (3.3) | 26.4 (3.7) | 25.9 (3.5) |
| **Mean (SD) donor age (years)** | 52.4 (14.0) | 65.1 (4.0) | 49.8 (12.0) | 50.6 (12.7) |
| **Donor ethnicity n (%)** | | | | |
| White | 7037 (94.2) | 716 (94.2) | 884 (89.1) | 521 (92.5) |
| Asian | 201 (2.7) | 28 (3.7) | 69 (7.0) | 27 (4.8) |
| Black | 92 (1.2) | 9 (1.2) | 24 (2.4) | 9 (1.6) |
| Other | 139 (1.9) | 7 (0.9) | 15 (1.5) | 6 (1.1) |
| **Donor diabetes n (%)** | 559 (7.6) | - | - | - |
| (missing) | 72 | - | - | - |
| **Donor cardiac disease n (%)** | 874 (12.0) | - | - | - |
| (missing) | 161 | - | - | - |
| **Median [IQR] donor creatinine at retrieval (umol/L)** | 88.0 [75.0 112.0] | 53.0 [45.0 60.0] | 54.0 [46.0 60.0] | 51.0 [43.0 57.0] |
| (missing) | 3781 | 0 | 0 | 0 |
| **Median [IQR] donor urine output within previous 24 hours (ml)** | 2430.0 [1587.0 3540.0] | - | - | - |
| (missing) | 2577 | - | - | - |
| **Donor IMD quintile n (%)** | | | | |
| 1 – Least deprived | 678 (18.5) | 199 (28.4) | 221 (24.4) | 100 (23.9) |
| 2 | 708 (19.3) | 170 (24.3) | 176 (19.4) | 86 (20.6) |
| 3 | 736 (20.0) | 139 (19.8) | 184 (20.3) | 79 (18.9) |
| 4 | 750 (20.4) | 115 (16.4) | 177 (19.5) | 87 (20.8) |
| 5 – Most deprived | 800 (21.8) | 78 (11.1) | 149 (16.4) | 66 (15.8) |
| (missing) | 3797 | 59 | 85 | 145 |
| **Recipient sex ratio (M:F)** | 4719:2750 | 473:287 | 635:357 | 279:284 |
| **Mean (SD) recipient height (cm)** | 169.9 (10.3) | 170.8 (10.3) | 171.4 (10.3) | 168.9 (10.0) |
| **Mean (SD) recipient weight (kg)** | 78.8 (16.6) | 76.9 (15.6) | 79.4 (16.2) | 76.1 (16.7) |
| **Mean (SD) recipient BMI (kg/cm^2)** | 27.2 (4.7) | 26.3 (4.3) | 26.9 (4.5) | 26.5 (4.6) |
| **Mean (SD) recipient age (years)** | 53.3 (13.3) | 51.7 (14.8) | 51.5 (12.4) | 50.7 (12.9) |
| **Recipient ethnicity n (%)** | | | | |
| White | 5233 (70.1) | 675 (88.8) | 855 (86.2) | 456 (81.0) |
| Asian | 1336 (17.9) | 54 (7.1) | 82 (8.3) | 66 (11.7) |
| Black | 727 (9.7) | 21 (2.8) | 41 (4.1) | 30 (5.3) |
| Other | 173 (2.3) | 10 (1.3) | 14 (1.4) | 11 (2.0) |
| **Recipient CMV n (%)** | | | | |
| Negative | 3293 (44.1) | 430 (56.6) | 506 (51.0) | 263 (46.7) |
| Positive | 4176 (55.9) | 330 (43.4) | 486 (49.0) | 300 (53.3) |
| **Recipient blood group n (%)** | | | | |
| A | 2947 (39.5) | 346 (45.5) | 410 (41.3) | 226 (40.1) |
| AB | 391 (5.2) | 18 (2.4) | 28 (2.8) | 30 (5.3) |
| B | 923 (12.4) | 81 (10.7) | 118 (11.9) | 82 (14.6) |
| O | 3208 (43.0) | 315 (41.4) | 436 (44.0) | 225 (40.0) |
| **Recipient high anti-HLA sensitisation n (%)** | 396 (5.3) | 32 (4.2) | 37 (3.7) | 65 (11.5) |
| **Recipient PRD n (%)** | | | | |
| Other | 4347 (58.2) | 482 (63.4) | 567 (57.2) | 351 (62.3) |
| Cystic Kidney Disease | 1368 (18.3) | 150 (19.7) | 249 (25.1) | 124 (22.0) |
| Diabetes | 1286 (17.2) | 79 (10.4) | 104 (10.5) | 58 (10.3) |
| Glomerulonephritis | 468 (6.3) | 49 (6.4) | 72 (7.3) | 30 (5.3) |
| **Recipient IMD quintile n (%)** | | | | |
| 1 – Least deprived | 1369 (18.3) | 206 (27.1) | 251 (25.3) | 140 (24.9) |
| 2 | 1358 (18.2) | 202 (26.6) | 247 (24.9) | 133 (23.6) |
| 3 | 1476 (19.8) | 158 (20.8) | 216 (21.8) | 116 (20.6) |
| 4 | 1575 (21.1) | 121 (15.9) | 152 (15.3) | 89 (15.8) |
| 5 – Most deprived | 1691 (22.6) | 73 (9.6) | 126 (12.7) | 85 (15.1) |
| **HLA mismatch group n (%)** | | | | |
| 1 | 574 (7.7) | 49 (6.5) | 0 (0.0) | 34 (6.0) |
| 2 | 2177 (29.1) | 122 (16.2) | 0 (0.0) | 108 (19.2) |
| 3 | 3810 (51.0) | 356 (47.3) | 0 (0.0) | 250 (44.4) |
| 4 | 908 (12.2) | 226 (30.0) | 992 (100.0) | 171 (30.4) |
| (missing) | 0 | 7 | 0 | 0 |
| **Transplant year** | | | | |
| 2010-12 | 1416 (19.0) | 143 (18.8) | 221 (22.3) | 69 (12.3) |
| 2013-15 | 1764 (23.6) | 212 (27.9) | 283 (28.5) | 155 (27.5) |
| 2016-18 | 2353 (31.5) | 239 (31.4) | 291 (29.3) | 178 (31.6) |
| 2019-21 | 1936 (25.9) | 166 (21.8) | 197 (19.9) | 161 (28.6) |
| **Median [IQR] cold ischaemia time (hours)** | 13.3 [10.3 16.8] | 3.7 [2.6 4.5] | 3.5 [2.4 4.5] | 5.5 [4.5 6.9] |
| (missing) | 34 | 35 | 38 | 8 |
| **Median [IQR] waiting time (years)** | 2.1 [1.0 3.5] | 0.7 [0.3 1.7] | 0.7 [0.3 1.5] | 1.4 [0.6 2.9] |
| (missing) | 17 | 264 | 315 | 28 |
| **Dialysis status at transplantation** | | | | |
| Not on dialysis | 1227 (16.4) | 322 (42.4) | 431 (43.4) | 134 (23.8) |
| Haemodialysis | 4660 (62.4) | 295 (38.8) | 399 (40.2) | 307 (54.5) |
| Peritoneal | 1581 (21.2) | 143 (18.8) | 162 (16.3) | 122 (21.7) |
| (missing) | 1 | 0 | 0 | 0 |
| **Outcome at end of follow-up period n (%)** | | | | |
| Alive with functioning graft | 5236 (70.1) | 590 (77.6) | 814 (82.1) | 465 (82.6) |
| Graft failure | 1115 (14.9) | 84 (11.1) | 76 (7.7) | 43 (7.6) |
| Death with functioning graft | 1118 (15.0) | 86 (11.3) | 102 (10.3) | 55 (9.8) |
| **Median [IQR] recipient creatinine (3 months)** | 140.0 [111.0 180.0] | 136.5 [115.0 164.0] | 123.0 [103.0 144.0] | 118.0 [96.0 142.0] |
| (missing) | 981 | 60 | 82 | 44 |
| **Median [IQR] recipient creatinine (12 months)** | 131.0 [105.0 168.0] | 131.5 [112.0 162.0] | 117.0 [98.0 139.0] | 114.0 [92.0 134.0] |
| (missing) | 1128 | 92 | 91 | 65 |
| **Median [IQR] recipient creatinine (60 months)** | 130.0 [103.0 172.0] | 136.0 [112.5 174.0] | 118.0 [97.0 141.0] | 113.0 [92.5 147.0] |
| (missing) | 3738 | 324 | 367 | 251 |

## Supplementary Table S3: Univariable associations with event times

Hazard ratios [95% confidence interval] for recipient, donor, and operative characteristics estimated from univariable Cox models with different events of interest using the ‘base case’. The ‘composite’ outcome is the first occurring of *either* graft failure or death with a functioning graft. Reference groups for categorical characteristics are the same as reported in Section S1. The reference group for ‘Dialysis status at transplant’ is ‘not on dialysis’. Human leukocyte antigen (HLA) mismatch levels are defined within the UK transplant registry as follows: ‘1’ indicates no mismatches; ‘2’ represents zero mismatches at the DR locus and zero or one at the B locus; ‘3’ includes cases with either zero mismatches at the DR locus and two at the B locus, or one mismatch at the DR locus and zero or one at the B locus; and ‘4’ includes cases with two mismatches at the DR locus, or one at the DR locus and two at the B locus. High anti-HLA sensitisation is defined as a recipient’s calculated reaction frequency exceeding 85%. BMI: Body mass index; CMV: Cytomegalovirus; PRD: Primary renal disease; IMD: Index of multiple deprivation.

| **Characteristic** | **Composite** | **Graft failure** | **Death without graft failure** |
| --- | --- | --- | --- |
| Recipient sex: Male | 1.038 [0.962, 1.120] | 0.932 [0.838, 1.036] | 1.162 [1.041, 1.298] |
| Recipient BMI | 1.019 [1.011, 1.027] | 1.014 [1.002, 1.025] | 1.024 [1.013, 1.036] |
| Recipient age | 1.030 [1.027, 1.033] | 0.998 [0.994, 1.001] | 1.076 [1.071, 1.082] |
| Recipient ethnicity: Asian | 1.072 [0.964, 1.191] | 1.208 [1.045, 1.396] | 0.945 [0.808, 1.104] |
| Recipient ethnicity: Black | 1.491 [1.309, 1.698] | 1.984 [1.684, 2.338] | 1.016 [0.817, 1.262] |
| Recipient ethnicity: Other | 0.827 [0.607, 1.127] | 0.988 [0.659, 1.481] | 0.674 [0.418, 1.089] |
| Recipient positive CMV | 1.211 [1.125, 1.305] | 1.151 [1.037, 1.277] | 1.277 [1.149, 1.419] |
| Recipient blood group: AB | 1.090 [0.914, 1.299] | 1.250 [0.988, 1.583] | 0.932 [0.714, 1.216] |
| Recipient blood group: B | 1.036 [0.917, 1.170] | 1.090 [0.920, 1.291] | 0.983 [0.825, 1.172] |
| Recipient blood group: O | 1.047 [0.965, 1.135] | 1.064 [0.949, 1.193] | 1.030 [0.919, 1.154] |
| Recipient high anti-HLA sensitisation | 1.096 [0.928, 1.294] | 1.095 [0.866, 1.383] | 1.096 [0.865, 1.390] |
| Primary renal disease: Cystic Kidney Disease | 0.693 [0.621, 0.773] | 0.572 [0.489, 0.670] | 0.852 [0.731, 0.993] |
| Primary renal disease: Diabetes | 1.874 [1.709, 2.056] | 1.133 [0.979, 1.312] | 2.906 [2.569, 3.286] |
| Primary renal disease: Glomerulonephritis | 0.995 [0.857, 1.155] | 0.917 [0.746, 1.127] | 1.102 [0.887, 1.368] |
| Recipient IMD quintile: 2 | 1.070 [0.948, 1.208] | 1.279 [1.072, 1.525] | 0.910 [0.769, 1.076] |
| Recipient IMD quintile: 3 | 1.117 [0.990, 1.259] | 1.236 [1.034, 1.476] | 1.026 [0.871, 1.209] |
| Recipient IMD quintile: 4 | 1.350 [1.201, 1.517] | 1.644 [1.388, 1.947] | 1.123 [0.955, 1.321] |
| Recipient IMD quintile: 5 – most deprived | 1.378 [1.226, 1.548] | 1.692 [1.429, 2.002] | 1.135 [0.965, 1.335] |
| Donor sex: Male | 0.983 [0.913, 1.058] | 1.009 [0.909, 1.119] | 0.958 [0.863, 1.064] |
| Donor BMI | 1.015 [1.006, 1.023] | 1.011 [0.999, 1.022] | 1.018 [1.007, 1.030] |
| Donor age | 1.026 [1.023, 1.029] | 1.019 [1.015, 1.024] | 1.033 [1.028, 1.037] |
| Donor ethnicity: Asian | 0.982 [0.812, 1.188] | 1.033 [0.796, 1.340] | 0.931 [0.706, 1.228] |
| Donor ethnicity: Black | 0.798 [0.582, 1.095] | 1.102 [0.753, 1.612] | 0.493 [0.279, 0.870] |
| Donor ethnicity: Other | 1.105 [0.833, 1.466] | 0.922 [0.599, 1.419] | 1.299 [0.893, 1.889] |
| Donor creatinine at retrieval | 1.002 [1.002, 1.003] | 1.002 [1.001, 1.003] | 1.002 [1.001, 1.003] |
| Donor IMD quintile: 2 | 0.960 [0.810, 1.138] | 1.000 [0.788, 1.269] | 0.921 [0.722, 1.174] |
| Donor IMD quintile: 3 | 1.014 [0.858, 1.198] | 1.005 [0.793, 1.274] | 1.023 [0.809, 1.294] |
| Donor IMD quintile: 4 | 0.970 [0.819, 1.150] | 1.017 [0.802, 1.289] | 0.924 [0.725, 1.178] |
| Donor IMD quintile: 5 – most deprived | 1.153 [0.979, 1.359] | 1.293 [1.031, 1.621] | 1.013 [0.797, 1.287] |
| Transplant year | 1.028 [1.014, 1.043] | 1.005 [0.986, 1.025] | 1.056 [1.034, 1.078] |
| HLA mismatch level: 2 | 1.195 [1.023, 1.396] | 1.489 [1.190, 1.862] | 0.943 [0.758, 1.172] |
| HLA mismatch level: 3 | 1.499 [1.298, 1.730] | 1.592 [1.288, 1.967] | 1.422 [1.170, 1.729] |
| HLA mismatch level: 4 | 1.257 [1.068, 1.479] | 1.235 [0.971, 1.571] | 1.281 [1.027, 1.597] |
| Living donor | 0.500 [0.457, 0.547] | 0.536 [0.473, 0.607] | 0.464 [0.408, 0.529] |
| Cold ischaemia time (hours) | 1.042 [1.036, 1.047] | 1.035 [1.027, 1.043] | 1.049 [1.040, 1.057] |
| Waiting time (years) | 1.104 [1.085, 1.124] | 1.081 [1.053, 1.109] | 1.127 [1.100, 1.155] |
| Dialysis status at transplant: Haemodialysis | 2.167 [1.955, 2.402] | 2.038 [1.763, 2.355] | 2.306 [1.990, 2.671] |
| Dialysis status at transplant: Peritoneal | 1.447 [1.276, 1.641] | 1.557 [1.310, 1.851] | 1.331 [1.107, 1.599] |

## Supplementary Table S4: Incidence and risk difference of graft failure

Average percentage incidence and difference in reported incidence of graft failure at one, three, five, and seven years (95% confidence interval) post-transplantation. Results are presented as the mean estimate with confidence intervals, calculated as mean ± 1.96 × standard deviation over 250 bootstraps. Analyses using multiple imputation have percentile-based confidence intervals (Section S4). HLA: Human leukocyte antigen; LDKT: Living donor kidney transplantation; DDKT: Deceased donor kidney transplantation. DDKT incidence changes between analyses due to IPTW reweighting using different subsets of LDKT.

| Analysis | LDKT incidence (%) | DDKT incidence (%) | Risk difference (%) |
| --- | --- | --- | --- |
| **'Base analyses', n = 10 915 transplantations** | | | |
| One year | 1.32 (0.85, 1.79) | 4.88 (4.38, 5.38) | 3.56 (2.86, 4.26) |
| Three years | 3.09 (2.36, 3.82) | 8.33 (7.69, 8.97) | 5.24 (4.24, 6.25) |
| Five years | 5.61 (4.60, 6.61) | 11.63 (10.82, 12.44) | 6.03 (4.71, 7.35) |
| Seven years | 8.30 (7.13, 9.48) | 15.66 (14.65, 16.67) | 7.36 (5.78, 8.94) |
| **Multiple imputation for key confounders, n = 26 624 transplantations** | | | |
| One year | 1.74 (1.68, 1.79) | 5.32 (5.26, 5.39) | 3.59 (3.48, 3.70) |
| Three years | 3.39 (3.31, 3.47) | 8.91 (8.83, 9.00) | 5.52 (5.37, 5.67) |
| Five years | 5.83 (5.63, 6.03) | 12.78 (12.66, 12.90) | 6.95 (6.66, 7.24) |
| Seven years | 8.69 (8.49, 8.90) | 17.07 (16.94, 17.20) | 8.37 (8.07, 8.67) |
| **COVID-19 era transplants excluded, n = 9319 transplantations** | | | |
| One year | 1.16 (0.70, 1.62) | 5.11 (4.57, 5.66) | 3.95 (3.24, 4.67) |
| Three years | 3.01 (2.31, 3.71) | 8.64 (7.94, 9.34) | 5.63 (4.67, 6.59) |
| Five years | 5.55 (4.55, 6.54) | 11.95 (11.07, 12.82) | 6.40 (5.03, 7.77) |
| Seven years | 8.30 (7.17, 9.44) | 16.00 (14.88, 17.13) | 7.70 (6.09, 9.31) |
| **HLA mismatch level included in propensity score model, n = 10 883 transplantations** | | | |
| One year | 1.24 (0.86, 1.63) | 4.84 (4.29, 5.39) | 3.60 (2.96, 4.24) |
| Three years | 3.10 (2.18, 4.01) | 8.28 (7.60, 8.96) | 5.19 (4.02, 6.35) |
| Five years | 5.50 (4.33, 6.67) | 11.76 (10.88, 12.64) | 6.26 (4.82, 7.71) |
| Seven years | 7.94 (6.56, 9.31) | 15.86 (14.78, 16.93) | 7.92 (6.24, 9.60) |
| **LDKT subgroup: Older living donors, n = 8229 transplantations** | | | |
| One year | 1.74 (0.72, 2.76) | 5.00 (4.54, 5.47) | 3.26 (2.10, 4.42) |
| Three years | 4.40 (2.66, 6.14) | 8.40 (7.77, 9.03) | 4.00 (2.17, 5.84) |
| Five years | 6.86 (4.48, 9.24) | 11.79 (11.01, 12.57) | 4.93 (2.38, 7.48) |
| Seven years | 11.52 (8.28, 14.76) | 15.67 (14.71, 16.62) | 4.14 (0.78, 7.50) |
| **LDKT subgroup: Poorest HLA-matched grafts, n = 8461 transplantations** | | | |
| One year | 1.01 (0.20, 1.82) | 4.97 (4.46, 5.49) | 3.96 (2.98, 4.95) |
| Three years | 3.36 (1.96, 4.75) | 8.39 (7.76, 9.03) | 5.04 (3.50, 6.57) |
| Five years | 6.05 (4.19, 7.92) | 11.77 (10.95, 12.60) | 5.72 (3.67, 7.77) |
| Seven years | 8.16 (6.01, 10.31) | 15.65 (14.66, 16.63) | 7.49 (5.10, 9.87) |
| **LDKT subgroup: KSS or altruistic living donors, n = 8032 transplantations** | | | |
| One year | 1.32 (0.36, 2.27) | 5.02 (4.53, 5.51) | 3.70 (2.61, 4.80) |
| Three years | 3.05 (1.46, 4.65) | 8.49 (7.80, 9.18) | 5.43 (3.65, 7.22) |
| Five years | 6.72 (3.99, 9.44) | 11.96 (11.15, 12.77) | 5.24 (2.36, 8.12) |
| Seven years | 9.05 (5.83, 12.27) | 15.82 (14.87, 16.78) | 6.78 (3.36, 10.19) |

## Supplementary Table S5: Incidence and risk difference of death with functioning graft

Average percentage incidence and difference in reported incidence (95% confidence interval) of death with functioning graft at one, three, five, and seven years post-transplantation. Results are presented as the mean estimate with confidence intervals, calculated as mean ± 1.96 × standard deviation over 250 bootstraps. Analyses using multiple imputation have percentile-based confidence intervals (Section S4). HLA: Human leukocyte antigen; LDKT: Living donor kidney transplantation; DDKT: Deceased donor kidney transplantation. DDKT incidence changes between analyses due to IPTW reweighting using different subsets of LDKT.

| Analysis | LDKT incidence (%) | DDKT incidence (%) | Risk difference (%) |
| --- | --- | --- | --- |
| **'Base analyses', n = 10 915 transplantations** | | | |
| One year | 1.11 (0.61, 1.60) | 2.50 (2.17, 2.84) | 1.39 (0.80, 1.98) |
| Three years | 3.85 (2.82, 4.88) | 5.44 (4.96, 5.92) | 1.59 (0.51, 2.66) |
| Five years | 7.11 (5.76, 8.45) | 9.60 (8.93, 10.26) | 2.49 (1.03, 3.94) |
| Seven years | 10.65 (9.02, 12.29) | 13.94 (13.03, 14.85) | 3.28 (1.43, 5.14) |
| **Multiple imputation for key confounders, n = 26 624 transplantations** | | | |
| One year | 1.16 (1.06, 1.25) | 2.44 (2.41, 2.47) | 1.28 (1.17, 1.40) |
| Three years | 3.97 (3.87, 4.07) | 5.53 (5.49, 5.58) | 1.56 (1.47, 1.65) |
| Five years | 7.76 (7.67, 7.84) | 10.01 (9.93, 10.10) | 2.26 (2.16, 2.36) |
| Seven years | 11.86 (11.65, 12.07) | 14.53 (14.44, 14.62) | 2.67 (2.43, 2.91) |
| **COVID-19 era transplants excluded, n = 9319 transplantations** | | | |
| One year | 0.98 (0.47, 1.49) | 2.20 (1.86, 2.55) | 1.23 (0.61, 1.85) |
| Three years | 3.67 (2.62, 4.72) | 5.06 (4.55, 5.56) | 1.38 (0.22, 2.55) |
| Five years | 6.83 (5.48, 8.18) | 9.19 (8.42, 9.97) | 2.36 (0.80, 3.93) |
| Seven years | 10.30 (8.64, 11.95) | 13.59 (12.62, 14.56) | 3.29 (1.36, 5.23) |
| **HLA mismatch level included in propensity score model, n = 10 883 transplantations** | | | |
| One year | 0.86 (0.44, 1.27) | 2.51 (2.15, 2.86) | 1.65 (1.13, 2.17) |
| Three years | 3.59 (2.52, 4.66) | 5.51 (4.98, 6.05) | 1.92 (0.74, 3.10) |
| Five years | 6.81 (5.41, 8.22) | 9.63 (8.89, 10.36) | 2.81 (1.25, 4.38) |
| Seven years | 10.47 (8.81, 12.14) | 14.32 (13.25, 15.39) | 3.85 (1.88, 5.82) |
| **LDKT subgroup: Older living donors, n = 8229 transplantations** | | | |
| One year | 1.44 (0.48, 2.41) | 2.76 (2.35, 3.17) | 1.31 (0.28, 2.35) |
| Three years | 5.38 (2.93, 7.83) | 6.01 (5.41, 6.61) | 0.63 (-1.83, 3.09) |
| Five years | 8.75 (5.79, 11.71) | 10.64 (9.85, 11.43) | 1.89 (-1.13, 4.92) |
| Seven years | 10.85 (7.63, 14.07) | 15.44 (14.49, 16.40) | 4.59 (1.29, 7.89) |
| **LDKT subgroup: Poorest HLA-matched grafts, n = 8461 transplantations** | | | |
| One year | 1.41 (0.44, 2.38) | 2.72 (2.38, 3.07) | 1.31 (0.27, 2.36) |
| Three years | 3.89 (2.20, 5.57) | 5.92 (5.37, 6.48) | 2.04 (0.34, 3.73) |
| Five years | 7.90 (5.72, 10.09) | 10.51 (9.72, 11.30) | 2.61 (0.30, 4.92) |
| Seven years | 10.91 (8.29, 13.54) | 15.23 (14.23, 16.24) | 4.32 (1.55, 7.09) |
| **LDKT subgroup: KSS or altruistic living donors, n = 8032 transplantations** | | | |
| One year | 1.52 (0.10, 2.94) | 2.77 (2.38, 3.15) | 1.25 (-0.26, 2.75) |
| Three years | 7.21 (3.07, 11.35) | 6.00 (5.47, 6.54) | -1.20 (-5.41, 3.00) |
| Five years | 9.81 (5.28, 14.35) | 10.67 (9.93, 11.41) | 0.86 (-3.86, 5.58) |
| Seven years | 12.62 (7.72, 17.53) | 15.41 (14.40, 16.43) | 2.79 (-2.26, 7.84) |

## Supplementary Table S6: Estimated coefficients from propensity score model

Estimated coefficients (SE: standard error) and odds ratios [95% Wald confidence interval] for parameters in the propensity score model (Section S1). High anti-HLA sensitisation is defined as a recipient’s calculated reaction frequency exceeding 85%. BMI: Body mass index; CMV: Cytomegalovirus; PRD: Primary renal disease; IMD: Index of multiple deprivation

| **Parameter** | **Estimate (SE)** | **Odds ratio [95% CI]** |
| --- | --- | --- |
| (Intercept) | 0.03 (0.08) | 1.03 [0.87, 1.21] |
| Recipient sex: Male | -0.09 (0.05) | 0.91 [0.83, 1.00] |
| Recipient BMI | -0.01 (0.00) | 0.99 [0.98, 1.00] |
| Recipient age | -0.03 (0.00) | 0.97 [0.96, 0.97] |
| Recipient ethnicity: Asian | -1.30 (0.09) | 0.27 [0.23, 0.32] |
| Recipient ethnicity: Black | -1.50 (0.12) | 0.22 [0.18, 0.28] |
| Recipient ethnicity: Other | -0.70 (0.17) | 0.50 [0.36, 0.69] |
| Recipient positive CMV | 0.03 (0.05) | 1.03 [0.94, 1.14] |
| Recipient blood group: AB | -0.28 (0.12) | 0.75 [0.60, 0.95] |
| Recipient blood group: B | 0.06 (0.08) | 1.07 [0.92, 1.24] |
| Recipient blood group: O | -0.04 (0.05) | 0.96 [0.88, 1.06] |
| Recipient high anti-HLA sensitisation | -0.25 (0.11) | 0.78 [0.63, 0.97] |
| Primary renal disease: Cystic Kidney Disease | -0.11 (0.06) | 0.90 [0.80, 1.00] |
| Primary renal disease: Diabetes | -0.17 (0.07) | 0.85 [0.73, 0.97] |
| Primary renal disease: Glomerulonephritis | -0.08 (0.09) | 0.93 [0.77, 1.11] |
| Recipient IMD quintile: 2 | 0.00 (0.07) | 1.00 [0.88, 1.14] |
| Recipient IMD quintile: 3 | -0.20 (0.07) | 0.82 [0.71, 0.93] |
| Recipient IMD quintile: 4 | -0.37 (0.07) | 0.69 [0.60, 0.79] |
| Recipient IMD quintile: 5 – most deprived | -0.67 (0.07) | 0.51 [0.44, 0.59] |
| Donor sex: Male | -0.52 (0.04) | 0.59 [0.54, 0.65] |
| Donor BMI | -0.01 (0.01) | 0.99 [0.98, 1.00] |
| Donor age | -0.01 (0.00) | 0.99 [0.99, 1.00] |
| Donor ethnicity: Asian | 1.86 (0.12) | 6.40 [5.10, 8.03] |
| Donor ethnicity: Black | 1.61 (0.18) | 5.00 [3.54, 7.05] |
| Donor ethnicity: Other | 0.29 (0.17) | 1.33 [0.95, 1.86] |
| Transplant year | -0.01 (0.01) | 0.99 [0.97, 1.00] |

## Supplementary Table S7: Associations between missing data and competing risks

Associations between the indicator for any missing data as well as for missingness in individual confounders in the propensity score model and the competing risk shown. $p$-values are estimated from log-rank tests using 26 624 transplantations. BMI: Body mass index; IMD: Index of multiple deprivation; CMV: Cytomegalovirus; cRF: Calculated reaction frequency (%), used to determine high anti-HLA sensitisation.

| Missingness indicator | Graft failure | Death with functioning graft |
| --- | --- | --- |
| Any missing confounder (n = 15709) | 0.004 | 0.217 |
| Primary renal disease (n = 8073) | <0.001 | 0.520 |
| Recipient BMI (n = 5454) | 0.001 | 0.191 |
| Recipient IMD (n = 4758) | 0.018 | 0.337 |
| Recipient CMV (n = 1263) | 0.022 | 0.120 |
| Donor BMI (n = 806) | 0.001 | 0.549 |
| Recipient ethnicity (n = 350) | <0.001 | 0.029 |
| Donor ethnicity (n = 153) | 0.061 | 0.075 |
| Recipient sex (n = 22) | 0.221 | 0.984 |
| Recipient blood group (n = 5) | 0.428 | 0.466 |
| Recipient cRF (n = 5) | 0.428 | 0.466 |

## Supplementary Figure S1: Flowchart of data exclusions and missing data


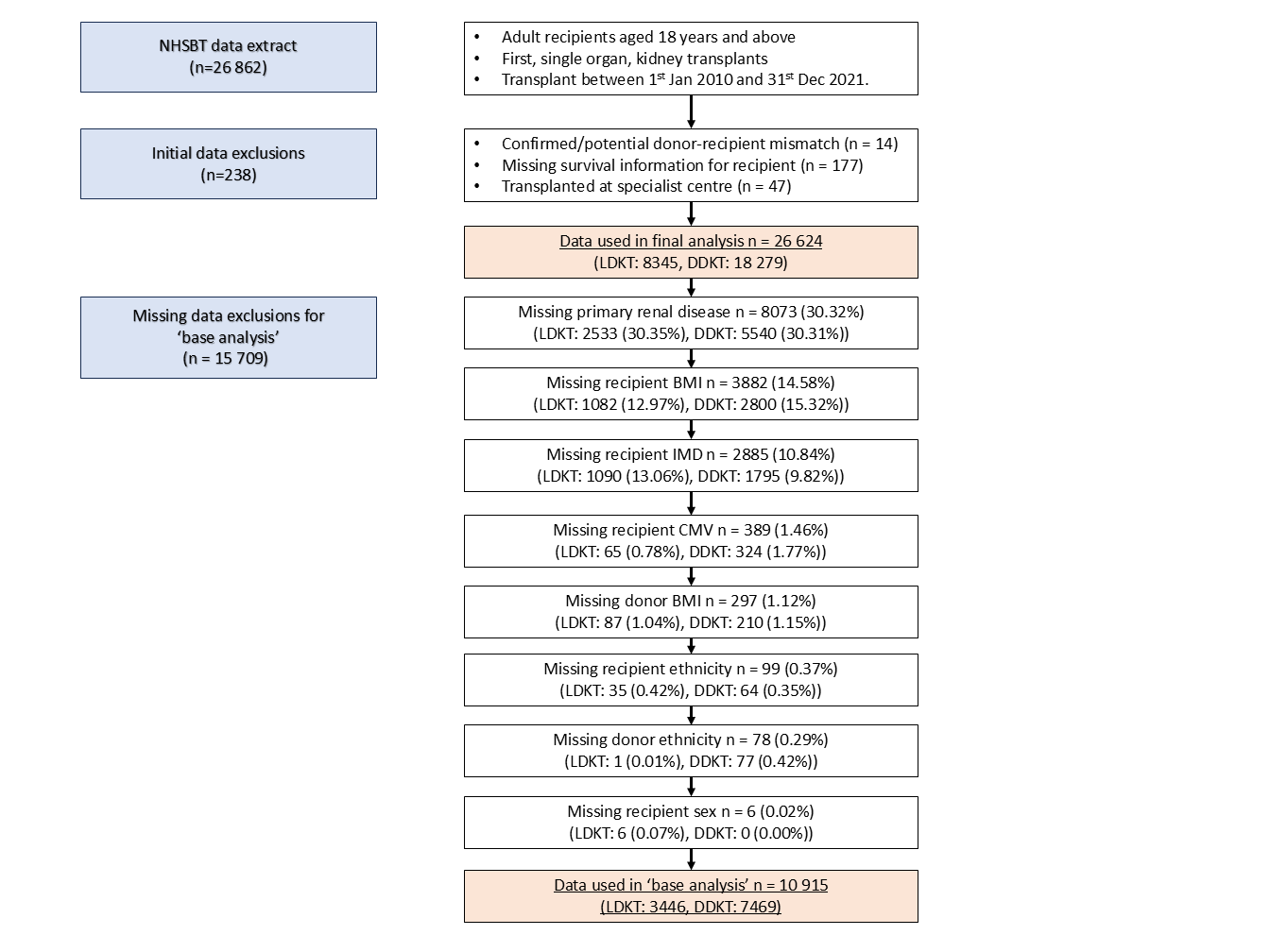
Flowchart summarising data exclusions from the NHS Blood & Transplant (NHSBT) data extract for analyses. Blue boxes represent data cleaning ‘stages’, and orange boxes represent analysis data sets. Percentages are based on the remaining 26 624 transplants after initial exclusions. Numbers removed at each stage are sequential, with all cases in one category (e.g., missing primary renal disease) excluded before proceeding to the next (e.g., missing recipient BMI). IMD: Index of multiple deprivation; CMV: Cytomegalovirus; BMI: Body mass index

## Supplementary Figure S2: Propensity score model – deviance residuals

Histogram of deviance residuals for the propensity score model (Section S1) split by donor type. The separation in the distributions is expected due to the binary outcome.


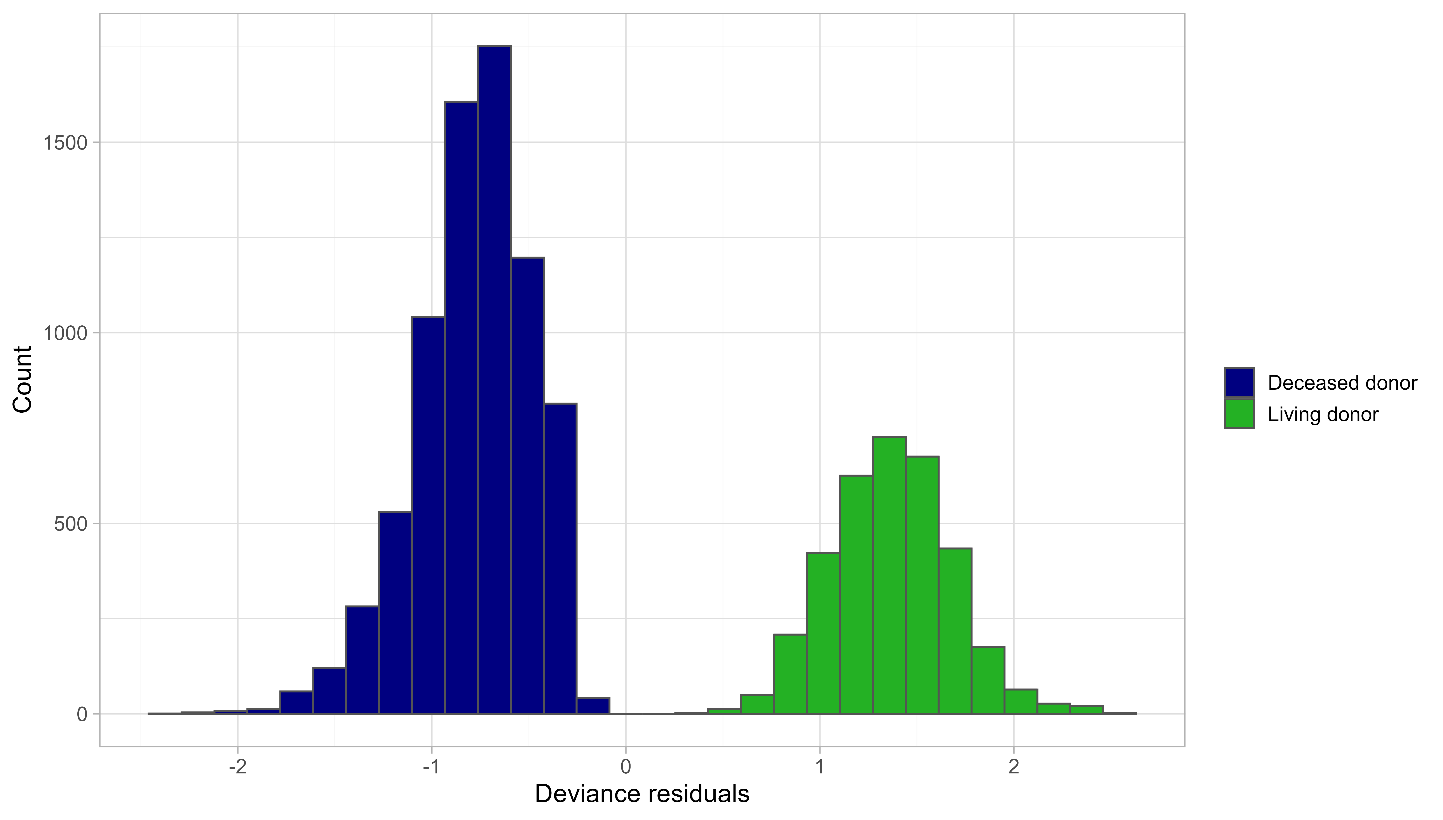


## Supplementary Figure S3: Propensity score model – distribution of predicted probabilities

Distribution of propensity scores $\hat{Pr}\left( Y_{i}=1 | R_{i},D_{i},O_{i} \right)$ estimated by the propensity score model (Section S1), split by donor type.


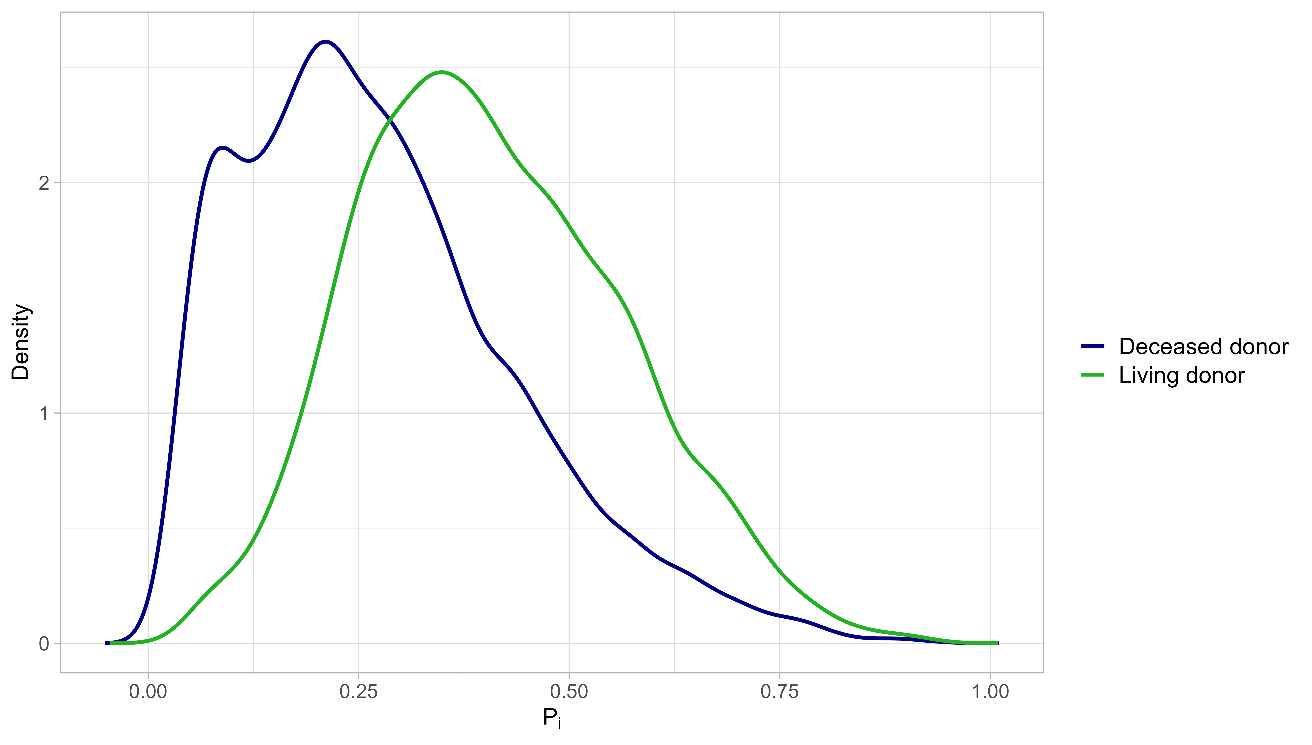


## Supplementary Figure S4: Patterns of missing data


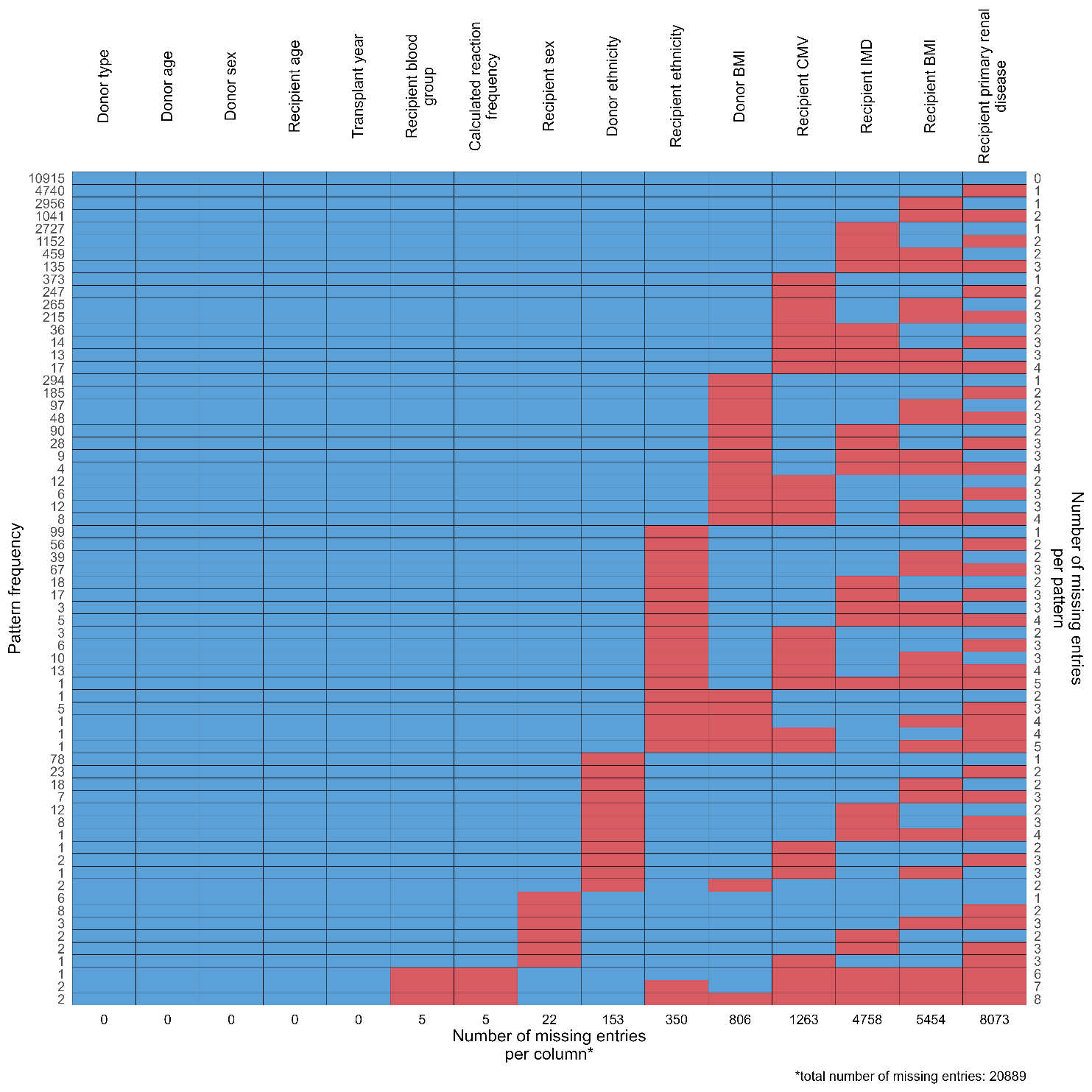
Missing data patterns for the key confounders in the propensity score model (Section S1), which form the columns. A (blue) red square indicates (no) missingness for the given confounder. The right-hand number indicates the number of missing covariates for each unique pattern of missingness; the left-hand number denotes the number of transplantations the missingness pattern is present for. That is, there are e.g. 4740 transplantations missing primary renal disease, and 1041 missing both primary renal disease and recipient body mass index (BMI). IMD: Index of multiple deprivation; CMV: Cytomegalovirus.

# References

Bartlett, J.W. and Hughes, R. A. (2020). Bootstrap inference for multiple imputation under uncongeniality and misspecification. *Statistical Methods in Medical Research* 29(12):3533-3546.
